# Salt-Enhanced Reproductive Development of *Suaeda salsa* L. Coincided With Ion Transporter Gene Upregulation in Flowers and Increased Pollen K^+^ Content

**DOI:** 10.3389/fpls.2019.00333

**Published:** 2019-03-29

**Authors:** Jianrong Guo, Xinxiu Dong, Guoliang Han, Baoshan Wang

**Affiliations:** Shandong Provincial Key Laboratory of Plant Stress, College of Life Sciences, Shandong Normal University, Jinan, China

**Keywords:** genes, ion homeostasis, NaCl, reproductive organs, *Suaeda salsa* L.

## Abstract

Halophytes are adapted to saline environments and demonstrate optimal reproductive growth under high salinity. To gain insight into the salt tolerance mechanism and effects of salinity in the halophyte *Suaeda salsa*, the number of flowers and seeds, seed size, anther development, ion content, and flower transcript profiles, as well as the relative expression levels of genes involved in ion transport, were analyzed in *S. salsa* plants treated with 0 or 200 mM NaCl. The seed size, flower number, seed number per leaf axil, and anther fertility were all significantly increased by 200 mM NaCl treatment. The Na^+^ and Cl^−^ contents in the leaves, stems, and pollen of NaCl-treated plants were all markedly higher, and the K^+^ content in the leaves and stems was significantly lower, than those in untreated control plants. By contrast, the K^+^ content in pollen grains did not decrease, but rather increased, upon NaCl treatment. Genes related to Na^+^, K^+^ and, Cl^−^ transport, such as *SOS1*, *KEA*, *AKT1*, *NHX1*, and *CHX*, showed increased expression in the flowers of NaCl-treated plants. These results suggest that ionic homeostasis in reproductive organs, especially in pollen grains under salt-treated conditions, involves increased expression of ion transport-related genes.

## Introduction

Salinity is an increasing problem worldwide, and can severely reduce crop growth and yield, particularly in irrigated land (Greenway and Munns, 1980; Munns, 2002; Rengasamy, 2006). Halophytes are plants that are adapted to saline soil environments (Flowers et al., 1977; Flowers and Yeo, 1986) and are able to survive and reproduce at salt concentrations of 200 mM or greater (Flowers and Colmer, 2008; Song and Wang, 2015; Guo et al., 2018), at which the yield of major crop plants is severely reduced. In the vegetative growth stage, euhalophyte survival mainly depends on the exclusion of Na^+^ and Cl^−^ and/or sequestration of these ions into vacuoles, which maintains ionic homeostasis and avoids toxicity in young, growing leaves (Wang et al., 2004; Han et al., 2005; Qiu et al., 2007; Munns and Tester, 2008; Yang et al., 2010). However, it is unclear whether the mechanism of salt tolerance in reproductive growth processes of euhalophytes is same as that in vegetative growth processes.

The growth and productivity of crops are markedly reduced by salt stress (Slama et al., 2015). Successful reproductive development is the limiting factor for crop yield, and reproductive growth is more sensitive than vegetative growth to environmental salt (Baby et al., 2016; Onyemaobi et al., 2017). Halophytes maintain ion homeostasis by actively controlling the uptake, storage, exclusion, and secretion of ions under saline conditions and exhibit maximal growth in both the vegetative and reproductive phases under high salinity (Flowers and Yeo, 1986; Li et al., 2011; Song et al., 2011, 2016; Chen et al., 2018; Guo et al., 2018). By contrast, the ability to maintain ionic homeostasis under salt stress is low in non-halophytes, such as crops (Durand and Lacan, 1994; Sahoo et al., 2016), and Na^+^ readily accumulates in the cytosol of such plants, with toxic effects (Forieri et al., 2016). Therefore, identifying the molecular mechanism underlying salt tolerance during the reproductive development of halophytes may present a strategy to generate crops that can withstand saline soil.

Flowering and seed formation are key events for the reproductive success of flowering plants, and a saline environment severely limits the reproductive growth and yield of non-halophytes (Khatun et al., 1995; Parvin et al., 2015). Several factors have been proposed to underlie the inhibition of growth and reproduction induced by salinity, including the accumulation of toxic ions such as Na^+^, K^+^ deficiency, plant hormone imbalance, and carbon supply reduction (Khatun and Flowers, 1995; Cuartero and Fernández-Muñoz, 1998; Munns, 2002; Plackett et al., 2011; Yang et al., 2017). In salt-sensitive chickpea (*Cicer arietinum*) plants, for example, treatment with 50 mM NaCl stimulates flower and pod abortion and reduces seed number (Kotula et al., 2015).

Anther and pollen development are crucial for male reproduction, and are coordinately regulated by many external and internal cues, which are highly sensitive to abiotic stresses (Sheoran and Saini, 1996; Oliver et al., 2005, 2007; Endo et al., 2009; Deng et al., 2012). A study by Grunberg et al. (1995) showed that the main cause of the salinity-induced decrease in tomato (*Solanum lycopersicum*) fruit yield is a reduction of pollen number rather than pollen fertility. In rice (*Oryza sativa*), however, the seed yield decreases under salinity have been found to be caused by the reduction of pollen fertility, and to be directly attributable to toxic ion accumulation in plants (Khatun and Flowers, 1995). In grapevine (*Vitis vinifera*), salinity negatively affects fruit set and is associated with poor pollen tube growth in the style (Baby et al., 2016). Furthermore, salinity has been reported to inhibit grain filling in wheat (*Triticum aestivum*) (Zheng et al., 2010) and reduce the receptivity of stigmas (Khan and Abdullah, 2003). However, only a few studies have examined flower and anther development of halophytes in saline environments. Flowering appears to be stimulated by the presence of NaCl in the halophyte *Plantago crassifolia*; however, the seed number is reduced because half of the spikes produce aborted seeds (Grigore et al., 2012). In the case of the edible halophyte *Crithmum maritimum*, salinity significantly reduces the numbers of inflorescences and flowering branches, and there are differences between genotypes in the timing of flowering initiation (Ventura et al., 2014).

*Suaeda salsa* is an annual herbaceous euhalophyte that grows in saline soil environments (Zhao et al., 2002), with an optimal salt concentration for both vegetative and reproductive growth of 200 mM NaCl (Pang et al., 2005; Zhang et al., 2005; Song et al., 2008; Guo et al., 2018). *S. salsa* is a promising model organism for studying salt tolerance mechanisms in euhalophytes, due to the availability of suitable genetic tools and plant materials, ease of growth, and short life cycle (Song and Wang, 2015). However, little is known about the molecular mechanisms underlying salt tolerance during reproductive growth in *S. salsa*, and elucidating these mechanisms could suggest a strategy to improve salt tolerance in crop species (Shi et al., 2003).

Ionic and pH homeostasis are basic regulators of plant growth and development. During vegetative growth, the Na^+^ content in the cytoplasm of salt-tolerant plants is reduced via efflux through Na^+^/H^+^ antiporters in the plasma membranes (Hasegawa, 2013). The intracellular Na^+^/H^+^ antiporter (NHX) family of proteins in vacuolar membranes plays an important role in sequestering Na^+^ or K^+^ into the vacuoles in plant cells under salt treatment (Munns and Tester, 2008; Barragán et al., 2012). Among these, NHX1 and NHX2 are essential regulators of intracellular K^+^ and pH homeostasis during flower development in Arabidopsis (*Arabidopsis thaliana*) (Bassil et al., 2011). The high-affinity K transporter (HKT) is responsible for the transport of Na^+^ and K^+^, and plays a crucial role in unloading Na^+^ from the xylem and maintaining a high intracellular K^+^/Na^+^ ratio in plant cells (Deinlein et al., 2014). For example, overexpression of *AtNHX1* enhanced the K^+^/Na^+^ ratio in tomato (Leidi et al., 2010). There are nine HKT proteins in rice, including OsHKT1;1, which is localized to anthers and functions in reproductive growth (Hamamoto et al., 2015). However, whether genes encoding proteins involved in ionic and pH homeostasis influence the reproductive growth of *S. salsa* is unknown.

The overall aim of this study was to identify which reproductive stages are markedly affected by NaCl and which genes are involved in reproductive processes in *S. salsa*. For this purpose, we subjected *S. salsa* plants to either control or optimum concentrations of NaCl (0 and 200 mM NaCl, respectively) in the sand medium from the time of sowing, and then monitored the plants throughout their growth period. We investigated reproductive parameters such as flower number, seed size, anther development, and ion content in leaves, stems, and pollen. We also analyzed the transcriptomes of *S. salsa* flowers from control and NaCl-treated plants using high-throughput Illumina RNA sequencing (RNA-seq). We then annotated the *S. salsa* reads against five protein and protein function databases and identified differentially expressed genes (DEGs) between the control and NaCl-treated groups. Next, we quantified the relative expression levels of genes involved in ionic homeostasis in flowers from control and NaCl-treated *S. salsa* plants using quantitative real-time PCR (qPCR). Our findings provide insight into the relationship between salt-tolerance traits and the reproductive development processes of halophytes, and thus may help to identify key factors specific to halophytes that are responsible for the changes in reproductive growth and seed set under saline conditions.

## Materials and Methods

### Plant Material and Growth Conditions

*Suaeda salsa* seeds were collected from the Yellow River Delta (37°20′ N; 118°36′ E) in Shandong, a central-eastern province of China, in 2016. The location is the same as that described by Guo et al. (2015). Seeds were stored in a refrigerator at temperatures of <4°C for at least 6 months prior to use.

Salt solutions were applied to the sand in which the seeds were sown, until seed maturity, as described by Guo et al. (2018). To maintain a relatively constant concentration of NaCl in the sand during the treatment time, volumes of 200 mM NaCl in Hoagland’s solution equivalent to three times the pot water-holding capacity were applied twice a day; non-saline control plants were treated similarly but using Hoagland’s solution without NaCl. There were 12 pots (two plants in each pot with a height of 30 cm and a diameter of 26 cm) for each concentration of NaCl. The plants were kept in a greenhouse under the natural light conditions of Shandong Normal University (36°40′ N; 117°00′ E), with average day/night temperatures of 28 ± 3/23 ± 3°C (day /night) and relative humidity of 60/80%.

### Vegetative Growth Assessment

Vegetative growth and the beginning of flowering were observed and recorded. At the end of vegetative growth [100 days after sowing (DAS)], the plant height and number of primary branches in each pot were measured. The aboveground parts of the plants (one seedling in each pot) were harvested from each of six replicate pots and washed with distilled water. Immediately after a quick drying using filter paper, the fresh weight (FW) of plant material was determined. Then the fresh material was dried in an oven (105°C for 10 min, followed by 80°C for 72 h) and the dry weight (DW) was measured.

### Reproductive Growth Assessment

#### Analysis of Flower and Seed Development

There is one inflorescence in each leaf axil in *S. salsa*. During the flowering period, the flowers on the branchlets and leaf axils at the same position (e.g., branch 1 versus branch 1, numbered from the bottom) from plants in the two treatment groups were counted, and the inflorescence diameter and flower diameter in each leaf axil were determined. Additionally, the seeds were hand-harvested for each individual plant at maturity and the seed size assessed.

#### Analysis of Anther Development

To assess the anther development of *S. salsa* from the control and 200 mM NaCl treatment groups, flowers beginning to open were selected randomly, and the development of anthers before flower opening was observed under a stereoscope (Nikon SMZ745T, Japan) and photographed until anther dehiscence. The total percentage of anther abortion was calculated as follows: [(number of aborted anthers)/total number of observed anthers] × 100%.

### Ion Quantification

At 95 DAS, the young leaves and corresponding stems of branches at the same position of plants from the two *S. salsa* treatment groups were collected for subsequent ion analysis. The collected materials were oven-dried as described previously and then used for ion determination. Twenty milligrams of oven-dried samples were cut into small pieces and extracted in a boiling water bath with 5 mL ultrapure water (Milli-Q Reference, Millipore, United States) for 10 h; subsequently, the solution was filtered and the final volume adjusted to 10 mL with more ultrapure water, and used for ion determination. The concentrations of Na^+^, K^+^, and Cl^−^ in dilutions of the extracts (using an extract dilution factor of 3 for Na^+^, K^+^, and Cl^−^ of the 200 mM NaCl treatment samples) were determined by ion chromatography, using a DIONEXICS-1100 instrument (Thermo, United States).

At 145 DAS (at the full-blossom period), pollen grains from the two *S. salsa* treatment groups were collected from 9:00 to 11:00 AM, and dried as described above. After the weight of samples measured, 0.1 g for each replicate was extracted in a boiling water bath with 5 mL ultrapure water (Milli-Q Reference, Millipore, United States) for 10 h. Subsequently, the solution was filtered and the final volume adjusted to 10 mL with more ultrapure water, and used for ion determination. The concentrations of Na^+^, K^+^, and Cl^−^ extracts (using a dilution factor of 3) were determined by ion chromatography (DIONEXICS-1100, Thermo, United States).

### Inflorescence Development of *S. salsa* Leaf Axils

To determine the effect of NaCl on the development of inflorescences from the leaf axils, we analyzed the flower and seed numbers in each leaf axil. The branches at the same position of *S. salsa* plants, numbered from the bottom, raised under four different conditions—0 mM NaCl (control), 200 mM NaCl, and two groups whose treatment was switched from 0 to 200 mM NaCl or vice versa at 104 DAS—were collected and then the flowers and seeds were observed and counted under a stereoscope (Nikon SMZ745T, Japan). The data included the flower number and seed number in each leaf axil from control and NaCl-treated plants, and the plants of the treatment conversion groups.

### Total RNA Extraction, Library Construction, and Sequencing

Flowers at an early developmental stage (before meiosis during anther development) were collected from control and NaCl-treated plants and were used for sequencing analysis. Three biological replicates of both the control and treated plants were used for RNA-seq. Total RNA was extracted from *S. salsa* flowers using a Total Plant RNA Extraction Kit (Karroten, Beijing, China) according to the manufacturer’s protocols, and the RNA integrity was assessed using the RNA Nano 6000 Assay Kit with the 2100 Agilent Bioanalyzer system (Agilent Technologies, Santa Clara, CA, United States). Total RNA and mRNA concentration were measured with a Qubit 2.0 Fluorometer (Life Technologies, Foster City, CA, United States) using a Qubit RNA Assay Kit.

Sequencing libraries were generated using the NEBNext Ultra^TM^ RNA Library Prep Kit for Illumina (NEB, United States) following the manufacturer’s recommendations and index codes were added to attribute sequences to each sample. Briefly, mRNA was purified from total RNA using poly-T oligo-attached magnetic beads. Fragmentation was carried out using divalent cations under elevated temperature in NEBNext First Strand Synthesis Reaction Buffer (5×). First-strand cDNA was synthesized using random hexamer primer and M-MuLV Reverse Transcriptase (RNase H^−^). Second-strand cDNA synthesis was subsequently performed using DNA Polymerase I and RNase H.

Sequencing was performed on a cBot Cluster Generation System using a TruSeq PE Cluster Kit v3-cBot-HS (Illumina) according to the manufacturer’s instructions. After cluster generation, the library preparations were sequenced on an Illumina Hiseq platform and paired-end reads were generated.

### Transcriptome Assembly and Gene Functional Annotation

Raw reads in fastq format were first cleaned using in-house perl scripts; low-quality reads and those containing a vector adapter sequence or poly-N were excluded. Furthermore, the Q20 and Q30 quality scores, GC content, and sequence duplication level of the clean data were calculated. *De novo* transcriptome assembly was performed using paired end reads that had passed the quality control test using Trinity (Grabherr et al., 2011) using default parameters, and was clustered using Corset (Davidson and Oshlack, 2014).

The assembled unigenes expressed in *S. salsa* flowers from control and NaCl-treated plants were annotated based on the Nr (NCBI non-redundant protein sequences) database (Pruitt et al., 2005), the COG (Cluster of Orthologous Group of proteins) database (Tatusov et al., 2000), KO (KEGG Ortholog) database (Kanehisa et al., 2008), Swiss-Prot (a manually annotated and reviewed protein sequence database) (Apweiler et al., 2004), and the Gene Ontology (GO) database (Ashburner et al., 2000) using BLAST searches. The raw data has been deposited in SRA database with Accession Nos. of SAMN09726234, SAMN09726235, SAMN09726236, SAMN09726237, SAMN09726238, and SAMN09726239. The assembled transcriptome has been deposited to the TSA database (Accession No. GHGG00000000).

### Differential Expression Analysis

Genes showing significantly different expression (DEGs) between the flowers of *S. salsa* plants with and without 200 mM NaCl treatment were detected using DESeq. The longest transcript among the different transcripts of the same gene from the splicing result was selected as a representative of the gene, and was used for subsequent analysis. And each “cluster” generated by Corset as a unigene. The *P*-values were adjusted to <0.05 to control for the false discovery rate using the Benjamini and Hochberg approach, and |log_2_(fold change)| > 2 was set as the threshold to judge the significance of gene expression differences.

### Expression Analysis of Genes Related to Ion Homeostasis Using qPCR

To analyze the relative expression levels of the DEGs involved in ion homeostasis between the flowers from control and NaCl-treated plants, quantitative real-time PCR (qPCR) was performed. Primers for qPCR of these genes were designed using Beacon Designer software (version 7.0) according to the cloned sequences (Supplementary Table S5). The actin gene (GenBank ID: EU429457) was used as an internal standard (Ma et al., 2009). Real-time PCR was performed with SYBR Green Dye (Takara, Dalian, China) using a real-time PCR platform (CFX96, Bio-Rad, Berkeley, CA, United States), as described in the instructions. The gene expression values were calculated using the 2^−ΔΔCT^ method (Livak and Schmittgen, 2001). Three biological replicates were performed in the present study.

### Statistical Analysis

The vegetative and reproductive growth assessments were performed randomly with six replicate plants, and the RNA-seq and qPCR analyses were performed with three replicates; the results are presented as means ± standard deviation (SD). The values were analyzed using SPSS (version 17) and one-way ANOVA statistical software packages. Different letters indicate significant differences among the means at the level of 0.05 according to Duncan’s test.

## Results

### NaCl Treatment Enhanced Vegetative Growth of *S. salsa*

At the end of vegetative growth (100 DAS), we determined the growth parameters of *S. salsa* cultured with 0 mM (control) and 200 mM NaCl. We found that the 200 mM NaCl treatment did not inhibit *S. salsa* growth; on the contrary, this treatment significantly increased the FW, dry weight, plant height, and primary branch number of *S. salsa*, by 67.6, 40.7, 13.3, and 12.3%, respectively, compared with the control plants (Supplementary Figure S1).

### NaCl Treatment Markedly Increased *S. salsa* Seed Number, Mean Mass, and Size

**FIGURE 1:**
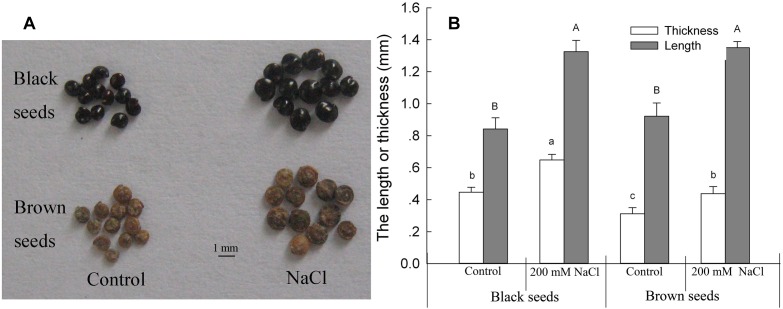
Black and brown seeds of *Suaeda salsa* plants. Photograph **(A)** and size **(B)** of seeds of plants grown in sand with a control nutrient solution (0 mM NaCl) and 200 mM NaCl treatment, respectively. Values are presented as the means ± SD of 100 replicates. Different letters indicate a significant difference at *P* < 0.05 according to Duncan’s test.

To investigate the differences between *S. salsa* seeds of the control and NaCl-treated plants, we hand-harvested the seeds from the individual plants at maturity and assessed their size. As reported in a previous study (Guo et al., 2018), NaCl treatment markedly increased the seed number per plant and the mean mass of individual seeds, as well as the seed size. It should be noted that *S. salsa* produces two different kinds of seeds (black seeds and brown seeds) on each plant, and the latter germinates faster and has more salt resistance than the former (Song et al., 2008; Guo et al., 2015). The length and thickness of black seeds and brown seeds from NaCl-treated plants were 125.9 and 128.0%, and 151.2 and 129.3%, respectively, of those from the control plants (Figure 1).

### NaCl Significantly Enhances *S. salsa* Leaf Axil Inflorescence Diameter and Flower Number

**FIGURE 2:**
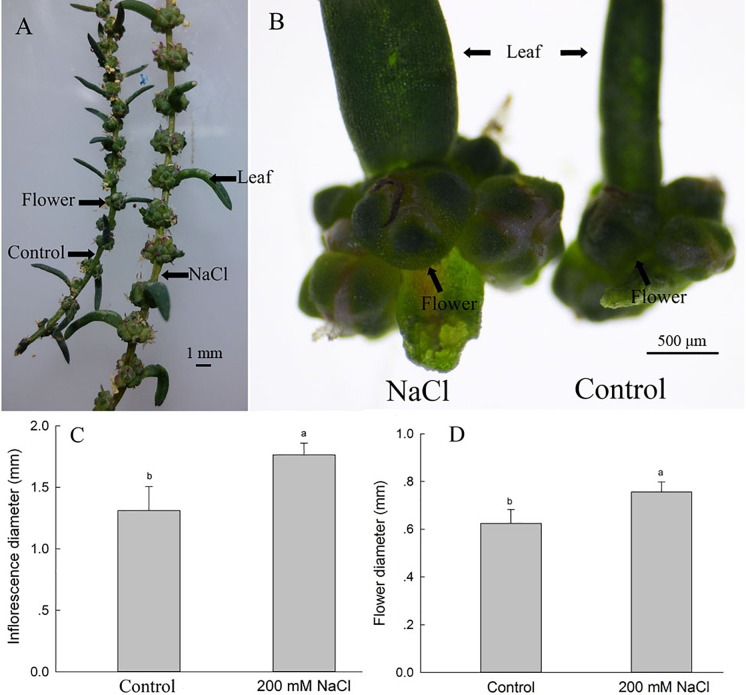
Inflorescence and flower parameters of *S. salsa* plants grown with and without 200 mM NaCl. Inflorescences of the same-numbered branchlet **(A)**, inflorescences in leaf axils **(B)**, inflorescence diameters of the leaf axils **(C)**, and flower diameters in the leaf axils **(D)** of *S. salsa* plants grown in sand medium with a control nutrient solution and with 200 mM NaCl treatment, respectively. Values are presented as the means ± SD of 50 replicates. Different letters indicate a significant difference at *P* < 0.05 according to Duncan’s test.

To investigate the possible reasons for the increased seed number and seed quality of *S. salsa* under saline conditions, we assessed the leaf axils from the branches at the same position of plants in the two groups, and found that 200 mM NaCl treatments significantly increased the number of flowers on the branchlets and leaf axils (Figure 2A,B); this increased flower number may be the basis for the increased number of seeds observed under 200 mM NaCl conditions. The diameters of the inflorescences and flowers were also increased in the 200 mM NaCl group, being 1.34- and 1.23-fold as large, respectively, as those of the control plants (Figure 2C,D), and thus also contributed to the increased seed size of NaCl-treated *S. salsa* plants (Figure 1).

### NaCl Profoundly Reduces *S. salsa* Anther Abortion

**FIGURE 3:**
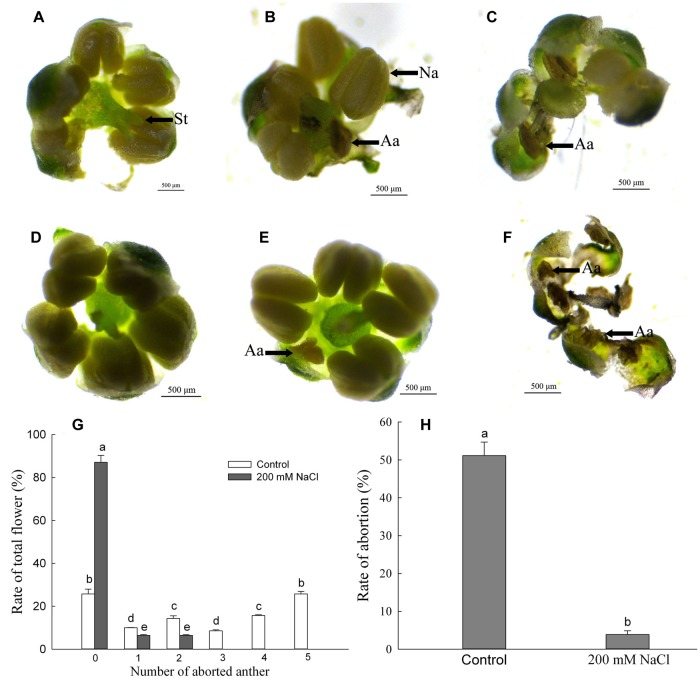
Anther development and rate of anther abortion of *S. salsa* plants grown in sand medium with a control nutrient solution (0 mM NaCl) and 200 mM NaCl treatment, respectively. **(A)** Normal flower from control plant. **(B)** Flower with one anther aborted from control plant. **(C)** Flower with three anthers aborted from control plant. **(D)** Normal flower from plant treated with 200 mM NaCl. **(E)** Flower with one anther aborted from plant treated with 200 mM NaCl. **(F)** Flower with five anthers aborted from control plant. **(G)** Rate of anther abortion of *S. salsa* plants. **(H)** Total rate of anther abortion of *S. salsa* plants. Values are presented as the means ± SD of 100 replicates **(G)** and 500 replicates **(H)**. Different letters indicate a significant difference at *P* < 0.05 according to Duncan’s test. St, stigma; Aa, abortive anther; Na, normal anther.

We collected random flowers from the branches at the same position on plants, numbered from the bottom, in the control and 200 mM NaCl treatment groups and observed them under a light stereoscope. Surprisingly, *S. salsa* anther development was significantly inhibited by the absence of NaCl; in the control plants, some of the anthers did not develop normally and the pollen production was reduced due to increased anther abortion (Figure 3B,C,F). Whereas *S. salsa* flowers normally contain five anthers per flower, flowers from the control plants contained from one to five aborted anthers, meaning that some flowers were likely to produce no pollen. The proportion of flowers with all five anthers aborted was 25.71% (Figure 3G). However, of the flowers of plants treated with 200 mM NaCl, only 12.91% contained aborted anthers, at a rate of only one or two per flower (Figure 3E,G)—significantly lower than the rate of anther abortion in the control plants. The proportion of normally developed flowers from control plants (Figure 3A) and from plants treated with 200 mM NaCl (Figure 3D) was 25.71% and 87.09% (Figure 3G), respectively. The total proportion of aborted anthers from the 200 mM NaCl treatment group was only 3.87%, significantly lower than that in the control group, which was 51.14% (Figure 3H).

### NaCl Is Beneficial to *S. salsa* Flower Bud Differentiation and Development

To evaluate which stages of the reproductive process of *S. salsa* were affected by salinity, we performed a treatment switch, in which the control plants were treated with 200 mM NaCl and the NaCl-treated plants with 0 mM NaCl up until seed maturation (defined as florescence of ∼50% leaf axils in which the first flower was opened, at 104 DAS). That is, the original control plants were switched to NaCl treatment (watered with Hoagland solution containing 200 mM NaCl; the 0→200 group), and the plants originally treated with 200 mM NaCl were switched to the control treatment (watered with regular Hoagland solution; the 200→0 group).

**FIGURE 4:**
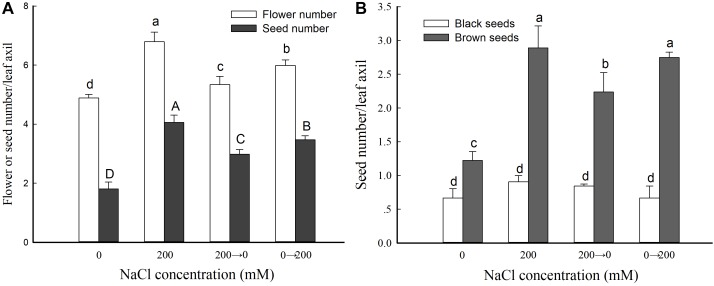
Flower and seed parameters in *S. salsa* grown in the presence and absence of 200 mM NaCl. Numbers of flowers and seeds **(A)** and numbers of black and brown seeds **(B)** in leaf axils of *S. salsa* plants grown in sand medium with a control nutrient solution (0 mM NaCl), 200 mM NaCl treatment, and the two switched treatments (0→200 NaCl and 200→0 NaCl). Values are presented as the means ± SD of 100 replicates. Different letters indicate a significant difference at *P* < 0.05 according to Duncan’s test.

We then analyzed the flower and seed numbers of leaf axils from the branches at the same position of plants from the four different treatment groups. Compared with the controls, the plants that received the 200 mM NaCl treatment throughout showed a significant increase in leaf axil flower number and seed number: 138.9 and 224.4% those of the control, respectively. The leaf axil flower number was 109.2 and 122.6% of the control in the 200→0 and 0→200 groups, respectively—higher than for the control but lower than for the 200 mM NaCl treatment group (Figure 4A). These results further indicated that exogenous NaCl treatment significantly promotes the reproductive growth of *S. salsa*, but not in the absence or removal of NaCl.

To further analyze the effect of NaCl on *S. salsa* seeds, we determined the numbers of black and brown seeds in flowers growing in the leaf axils (Figure 4B). The number of brown seeds was significantly increased by NaCl treatment (to 236.5% that of the control). Moreover, the brown seed number was 182.9 and 225.0% that of the control for the plants switched from NaCl to control conditions (200→0) and from control to NaCl conditions (0→200), respectively. However, there was no difference in black seed number between the different treatments (Figure 4B).

### NaCl Treatment Increases Na^+^ and Cl^−^ and Decreases K^+^ in Leaves and Stems, but Not in Pollen Grains

**FIGURE 5:**
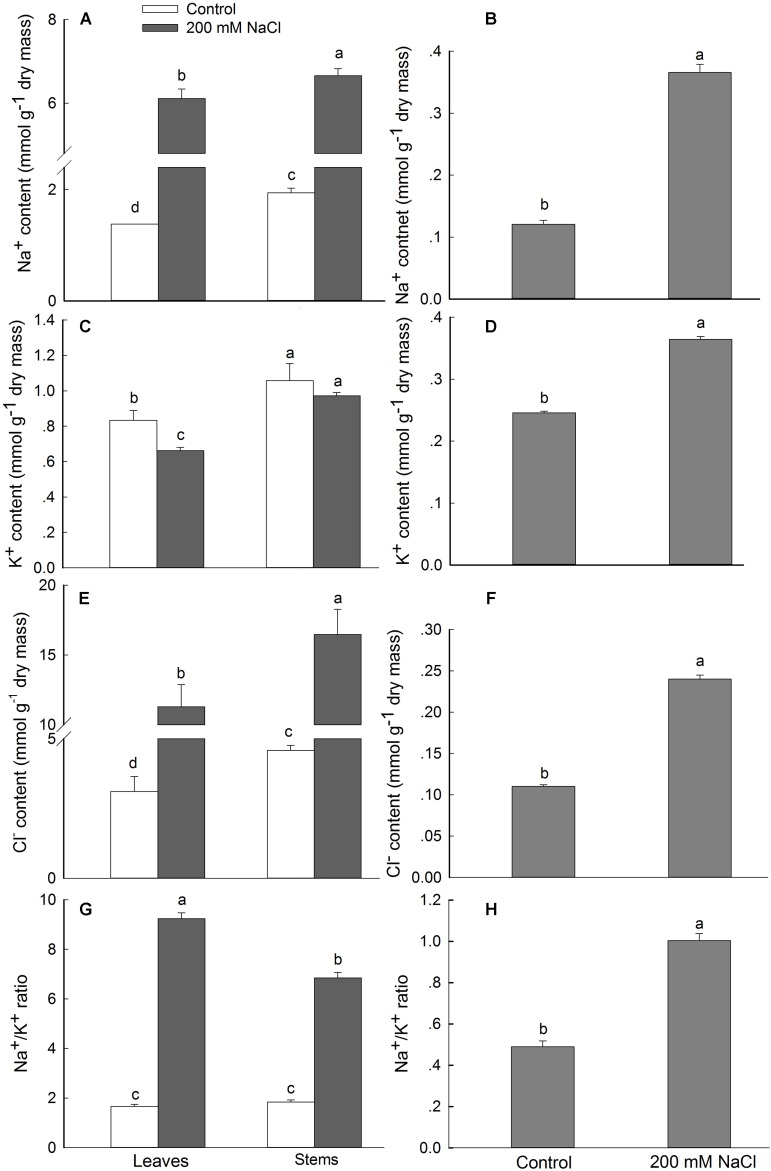
Ion contents of *S. salsa* plants grown in the presence or absence of 200 mM NaCl. The contents of Na^+^ **(A,B)**, K^+^ **(C,D)**, and Cl^−^ **(E,F)** and the Na^+^/K^+^ ratio of the leaves, stems, and pollen of *S. salsa* plants grown in sand with a control nutrient solution (0 mM NaCl) and 200 mM NaCl treatment at the end of vegetative growth (100 DAS; **A,C,E,G**) and at the flower stage (145 DAS of pollens; **B,D,F,H**). Values are presented as the means ± SD of four replicates. Different letters indicate a significant difference at *P* < 0.05 according to Duncan’s test.

Ion analysis showed that NaCl treatment induced a significant increase in Na^+^ and Cl^−^ contents in the leaves and stems at the end of the vegetative growth stage (Figure 5A,E), and that the Na^+^ and Cl^−^ content in flowers at the early flower stage was also significantly increased (Guo et al., 2018). The mean Na^+^ contents in the leaves and stems at the end of vegetative growth in the 200 mM NaCl treatment group were 6.12 and 6.66 mmol g^−1^ dry mass, which were 4.43- and 3.42-fold the values for the control group, respectively (Figure 5A). During reproductive growth, the flowers of NaCl-treated plants also accumulated significantly more Na^+^ than those of the control plants (Guo et al., 2018), and much less Na^+^ accumulated in the pollen than in the leaves and stems (Figure 5B). Similar trends were observed for Cl^−^ content in the leaves and stems of *S. salsa* when plants were treated with 200 mM of NaCl (Figure 5E), and a much smaller increase in Cl^−^ content was observed in the pollen than in the vegetative organs for the NaCl-treated plants as compared with control plants (Figure 5F).

By contrast, the K^+^ content of these organs was reduced significantly under NaCl treatment, except in the stamens and pistils (Guo et al., 2018) and in the pollen grains at the flower stage (Figure 5D). A highly significant (*P* ≤ 0.05) decrease in K^+^ content in leaves (20.52% reduction) and a smaller reduction in the stems (8.10%) was observed at the end of the vegetative growth stage of *S. salsa* treated with NaCl (Figure 5C). Interestingly, the K^+^ content in the pollen was observably higher (1.48-fold) than that in the control (Figure 5D). NaCl treatment significantly increased the Na^+^/K^+^ ratio in leaves and stems at the end of the vegetative growth stage, to 5.57- and 3.73-fold those from the control, respectively (Figure 5G). In the pollen grains, the Na^+^/K^+^ ratio for plants treated with NaCl was also observably higher than that for the control plants, despite the high K^+^ content in the pollen grains (Figure 5H). These results indicated that a certain Na^+^ content and a certain Na^+^/K^+^ ratio in the flowers and pollen grains of *S. salsa* are needed for proper development of the reproductive organs. These findings suggest that the euhalophyte *S. salsa* maintains ion homeostasis, particularly K^+^ and Na^+^ homeostasis, during pollen development under saline conditions.

### Sequencing Output and Assembly

To evaluate the molecular mechanisms underlying the improved reproduction in *S. salsa* in the presence of NaCl, we collected flowers from control and NaCl-treated plants and subjected them to RNA-seq. The total number of the raw and clean reads obtained from the NaCl and control groups is shown in Supplementary Table S1. The clean read data were then used to generate a *de novo* transcriptome assembly with Trinity (Grabherr et al., 2011), then was clustered using Corset (Davidson and Oshlack, 2014), and the differences in gene expression between the two groups were analyzed. The obtained transcripts and genes were shown in Supplementary Tables S2, S3. Among them there are 24,539 genes included a complete ORF. The number of obtained genes annotated to NR, NT, KO, SP, PFAM, GO, and COG database was shown in Supplementary Table S4. The integrity was 89.1% in the assembled transcriptome using BUSCO (3.0.2) software, among which 25.6% was the complete and single-copy BUSCOs, and 50.1% was complete and duplicated BUSCOs.

### Analysis of Differentially Expressed Genes (DEGS) Between Control and NaCl Groups

**FIGURE 6:**
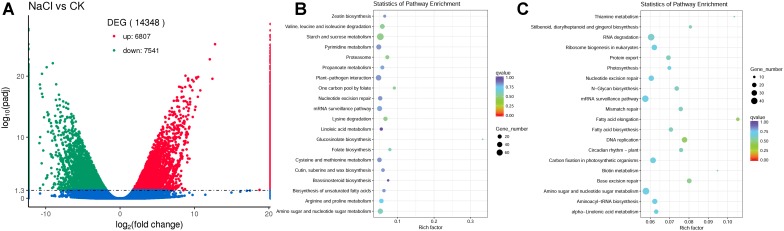
Number of differentially expressed genes (DEGs) between control and NaCl-treated plants **(A)**, the upregulated DEGs enriched top 20 KEGG pathway **(B)** and the downregulated DEGs enriched top 20 KEGG pathway **(C)** in *S. salsa* flowers subjected to the NaCl treatment; DEGs between the control and NaCl-treated group were assigned to KEGG pathways using enrichment statistics. In the scatterplot, each dot indicates a single gene. Blue dots represent genes with no significant difference in expression between the NaCl and control treatments; red dots represent significantly upregulated genes; and green dots represent significantly downregulated gene.

To evaluate the differences in gene expression in the flowers between control and NaCl-treated plants, the software package RSEM (Li and Dewey, 2011) was used to quantify the RNA-Seq data, and DESeq2 (Love et al., 2014) was used for differential expression analysis. We identified 14,348 genes that showed differential expression in flowers between control and NaCl-treated *S. salsa* plants. Among these DEGs, 6807 were upregulated and 7541 were downregulated by NaCl treatment (Figure 6A).

To gain insight in the possible functions of these genes and their utilities in biological systems, we further analyzed the DEGs using the GO (Ashburner et al., 2000) database (Supplementary Figure S2) and the KEGG (Kanehisa et al., 2008) database (Figure 6B,C). A total of 8385 DEGs were assigned to GO terms, of which 3773 DEGs genes were upregulated and 4612 DEGs were downregulated, and the terms with most significant difference were shown in Supplementary Figure S2. Based on the KEGG database, there were 3640 DEGs assigned to 124 KEGG pathways, the top 20 KEGG pathway that the upregulated DEGs enriched and the top 20 KEGG pathway that the downregulated DEGs enriched were shown in Figure 6B,C. And these metabolic pathways may be closely related to the reproductive development of *S. salsa* under NaCl treatment. In the upregulated genes, the majority of them were mapped to different pathways, for example, “starch and sucrose metabolism” category, in which 78 DEGs were upregulated and 55 DEGs were downregulated; “biosynthesis of unsaturated fatty acids” category, in which 11 DEGs were upregulated and 8 DEGs were downregulated; “zeatin and brassinosteroid biosynthesis” category, in which 12 DEGs were upregulated and 5 DEGs were downregulated. And in the downregulated genes, a lot of DEGs were mapped to “carbon fixation in photosynthetic organisms” category, in which 19 DEGs were upregulated and 33 DEGs were downregulated. It indicated that NaCl treatment mainly changed carbon fixation, carbohydrate metabolism and hormone biosynthesis in flowers of *S. salsa*. The reproductive growth process of *S. salsa* was promoted when treated with NaCl, and this promotion was inseparable from the related DEGs. Under salt treatment conditions, all the growth and metabolism of plants are based on the establishment of ion homeostasis. Once the ion homeostasis is destroyed, it will inevitably lead to the disturbance of growth and metabolism. Therefore, the increased growth and metabolism of *S. salsa* treated with NaCl was inseparable from the ion homeostasis in the reproduction organs. And the list of all identified DEGs in *S. salsa* flowers between control and NaCl-treated groups were shown in Supplementary Table S6.

### DEG_S_ Related to Ion Homeostasis in *S. salsa* Flowers

Ion homeostasis and pH homeostasis of plants is considered to be critical for plant growth and survival in a saline environment. These processes require the proton-motive force (PMF) (mainly produced by plasma membrane H^+^-ATPases, V-ATPase, and V-PPase), as well as ion transporters in the membrane. Table 1 lists the DEGs in *S. salsa* flowers encoding proteins involved in ion transport, which include cation/H^+^ exchangers, chloride transporters, potassium transporters, H^+^-ATPases, and H^+^-pyrophosphatases. Genes encoding cation/H^+^ exchangers were significantly upregulated in *S. salsa* flowers when the plants were treated with NaCl. Five cation/H^+^ exchanger genes as well as a gene encoding an Na^+^/H^+^ antiporter in the plasma membrane (SOS1) were expressed threefold or more in the flowers of *S. salsa* treated with NaCl. Furthermore, the expression of genes encoding Cl^−^ channels and carriers, such as ICln and CLC, was also increased by NaCl treatment. On the other hand, the expression of the genes encoding proteins involved in K^+^ uptake, such as KEA and AKT, was also increased many-fold by NaCl treatment. Furthermore, genes encoding H^+^-transporting ATPases (in the plasma membrane and tonoplast) and H^+^-pyrophosphatases (in the tonoplast), which provide energy for ion transport, were all significantly upregulated in the flowers of *S. salsa* plants treated with NaCl.

**Table 1:** List of upregulated genes in NaCl-treated *S. salsa* flowers determined by RNA-seq analysis; these genes are related to ion transport and proton-motive force.

Gene ID	Control readcount	NaCl readcount	log_2_FC	Regulated
Cation/hydrogen exchanger				
Cluster-10319.9702 (SOS1)	0	1877.190742	Inf	Up
Cluster-10319.8664 (CHX14)	0	297.5807	Inf	Up
Cluster-10319.135147 (CHX13)	10.09135025	116.5782369	3.5301	Up
Cluster-10319.51233 (CHX28)	33.99452	389.5622	3.5185	Up
Cluster-10319.95871 (NHX1)	25.61988	1100.805	5.4252	Up
Chloride channel				
Cluster-10319.165251 (ICln)	0	57.07504589	Inf	Up
Cluster-10319.105320 (CLC)	0	40.91814273	Inf	Up
Potassium transporter				
Cluster-10319.107095 (KEA3)	0	21.35734921	Inf	Up
Cluster-10319.125508 (KEA5)	0	17.01548	Inf	Up
Potassium channel				
Cluster-10319.47800 (AKT1)	0.299214025	63.78367483	7.7359	Up
H^+^-transporting ATPase				
Cluster-10319.76030 (AHA4)	0	29.03297191	Inf	Up
Cluster-10319.82266 (VHA)	2.094498173	280.6502929	7.066	Up
H^+^-pyrophosphatase				
Cluster-10319.102050 (AVP1)	0	21.22483451	Inf	Up

*Significantly differentially expressed genes were filtered using the thresholds of q < 0.005 and |log_2_(FC)| > 1. log_2_FC (log_2_ fold change) equals log_2_ (NaCl read count/control read count); up indicates that a gene was upregulated.*

### Relative Expression Levels of Genes Related to Ion Homeostasis

**FIGURE 7:**
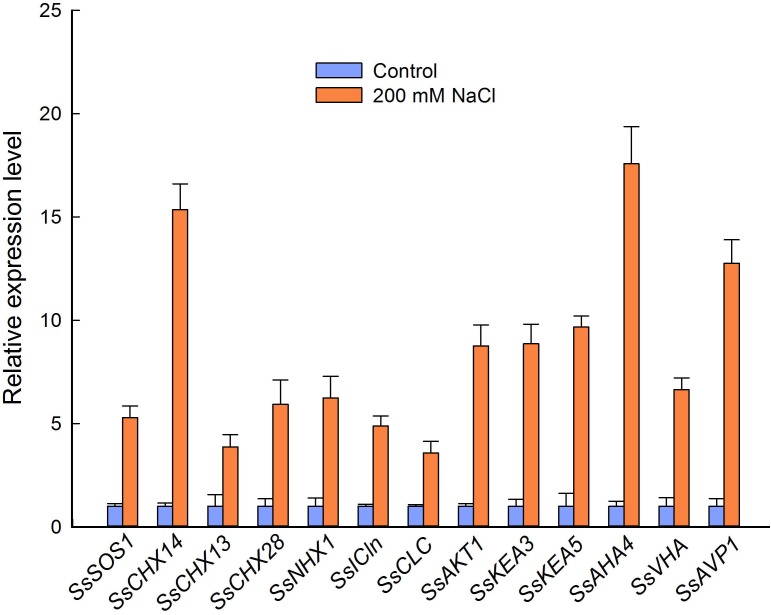
Relative expression level of genes encoding cation/H^+^ exchangers, Cl^−^ channels, K^+^ transporters, and proton pumps in the flowers of *S. salsa* plants grown in sand with a control nutrient solution (0 mM NaCl) and 200 mM NaCl. Values are presented as the means ± SD of three biological replicates.

To further analyze the relationship between ion content and the expression level of genes related to ion homeostasis in flowers from control and NaCl-treated plants, we selected 13 genes encoding Na^+^, K^+^, and/or Cl^−^ transporters or channels and the membrane-bound proton pumps related to the PMF, according to the RNA-seq results. We then performed qPCR based on the partial sequences of the genes obtained by regular PCR. NaCl treatment significantly increased the relative expression level of genes encoding proteins involved in ion homeostasis and pH homeostasis in *S. salsa* flowers, such as *SsSOS1*, *SsNHX1*, *SsAKT1*, and *SsCLC*. Strikingly, genes encoding a cation/H^+^ exchanger (CHX14), PM-H^+^-ATPase (AHA4), and V-H^+^-PPase (AVP1) showed very high relative expression levels in the flowers of NaCl-treated plants (15.3, 17.6, and 12.7 times those in the control plants, respectively) (Figure 7). Genes encoding K^+^ channels or transporters (*SsAKT1*, *SsKEA3*, and *SsKEA5*) and Cl^−^ channels (*SsICln* and *SsCLC*) were also significantly upregulated in flowers subjected to NaCl treatment, with expression levels 8.7, 8.9, 9.7, 4.9, and 3.6 times those in control plants, respectively (Figure 7).

## Discussion

High salinity frequently decreases the vegetative growth of glycophytes, but improves the growth of halophytes (Munns and Tester, 2008; Song et al., 2009; Song and Wang, 2015). The results presented here confirm previous observations that the vegetative growth of the halophyte *S. salsa* is positively affected by salt treatment, as this plant displayed enhanced growth in the presence of 200 mM NaCl (Supplementary Figure S1). This agrees with a previous report that the total biomass and photosynthesis rate of *S. salsa* are greater when the plants are grown in medium containing 200 mM NaCl than in medium containing 0 or higher NaCl concentrations (Lu et al., 2003; Pang et al., 2005; Zhang et al., 2005; Qiu et al., 2007). Growth in 200 mM NaCl provides more photoassimilates for the reproductive organs such as anther development than the control.

The reproductive processes of non-halophytes are considered to be salt sensitive (Khatun et al., 1995; Vadez et al., 2007, 2012; Samineni et al., 2011; Turner et al., 2013; Deng et al., 2016). Salinity largely inhibits reproductive organ formation (Li et al., 2007), decreases fertility (Amzallag, 2005), and reduces pollen tube growth in the style (Baby et al., 2016); thus, it also reduces seed number (Kotula et al., 2015). For example, in Arabidopsis, NaCl (200 mM) stress for 4 h resulted in a statistically insignificant reduction in fertility, but when maintained for longer than 12 h it resulted in a maximal decrease in reproduction, such that only 5% of ovules formed seeds (Sun et al., 2004). NaCl treatment also greatly reduced growth and seed production in chickpea (Sohrabi et al., 2008). By contrast, in the euhalophyte *S. salsa*, NaCl treatment significantly increased the total seed yield and the seed number as compared with the control (Guo et al., 2018), which implies that the presence of high concentrations of NaCl improves seed development in *S. salsa*, increasing the seed number and individual seed mass; 200 mM NaCl in the growth medium seems to be the optimal condition for reproductive growth (Guo et al., 2018).

In plants, stress-induced male sterility generally has a negative effect on crop yield and performance (Storme and Geelen, 2014). For example, drought inhibits starch accumulation in pollen and leads to male sterility in rice (Nguyen et al., 2010). Low temperature during the induction period reduces the amount of pollen in rice (Noctor, 2015), and the pistils are relatively less sensitive than the stamens (Loka and Oosterhuis, 2014). In glycophytes, unexpected conditions during male gametophyte development are often associated with dramatic yield losses (Pacini and Dolferus, 2016). However, for halophytic *S. salsa*, we discovered here that the development of anthers was much more efficient in plants treated with 200 mM NaCl than in control plants not treated with additional NaCl. In the presence of 200 mM NaCl, the total anther abortion ratio was only 3.87% (Figure 3H), and only 1–2 anthers were aborted per affected flower. By contrast, the total anther abortion ratio in control plants reached 51.14% (Figure 3H), and the proportion of flowers with all five anthers aborted in one flower was also high (Figure 3G). Therefore, anther development was markedly inhibited when *S. salsa* plants were grown in nutrient solution without additional NaCl, resulting in decreases in seed number and seed weight (Guo et al., 2018). This is opposite to the response of non-halophyte plants treated with salt.

The leaf axils of *S. salsa* are indefinite inflorescences: the flower at the center of leaf axils appears first, and then more flowers are produced at its flanks. To a certain extent, seed development in the leaf axils can reflect the seed development conditions of the whole plant. To investigate which stage of *S. salsa* reproductive biology was affected by salt, we determined the flower and seed number of leaf axils in plants that received the 0 mM NaCl control treatment, 200 mM NaCl, NaCl switched to control (200→0 group), and control switched to NaCl (0→200 group). The number of flowers in leaf axils in the 0→200 group was significantly increased compared with control and 200→0 (Figure 4A). This indicated that NaCl improves flower differentiation in the axils of *S. salsa*.

Analysis of the numbers of black and brown seeds in flowers growing in the leaf axils indicated that NaCl treatment increased the seed number in the leaf axils mainly by increasing the flowers’ fertility and the brown seed number, but not the black seed number (Figure 4B). These results indicate that the presence of salt during reproductive growth can improve the flowering and seed production of *S. salsa*. Thus, the reproductive growth (flower differentiation and seed development) of *S. salsa* requires a certain concentration of external NaCl. By contrast, the reproductive growth of non-halophytes is greatly limited by NaCl (Kotula et al., 2015).

In order to explore the molecular mechanisms of *S. salsa* reproduction improved by NaCl, RNA-seq was used to reveal differential gene expression between control and NaCl treated groups. A large number of genes (219,073) and a lower number of ORF representations (24,539) were obtained in the transcriptome of *S. salsa*. Perhaps, the high number of duplicated BUSCO coverage (50.1%) suggested the possibility of that *S. salsa* is a polyploidy, just like in quinoa, which is in the same Caryophyllales (Jarvis et al., 2017). Another possibility is that 24,539 genes with complete ORFs more likely represent the number of protein-coding genes and the remaining ∼180,000 “genes” most likely non-coding RNAs in *S. salsa*. But it needs of a further study to confirm.

Ion homeostasis of plants in saline environments is considered to be crucial for plant growth and survival. The yield decrease of crop plants under high salinity is mainly due to the accumulation of Na^+^ and Cl^−^ in reproductive structures (Samineni et al., 2011). Khatun and Flowers (1995) have suggested that the poor pollen viability observed in rice under saline conditions is caused by Na^+^ and Cl^−^ accumulation in both pollen and stigmas, which reduces seed set. However, this is not the case for the euhalophyte *S. salsa*, in which NaCl treatment significantly increased the Na^+^ and Cl^−^ content in the stamen and pistil (Guo et al., 2018) and in pollen grains (Figure 5B,F), whereas the K^+^ content increased (Figure 5D). This was quite different from the trend seen in leaves and stems, in which the K^+^ content significantly decreased in conjunction with the increased Na^+^ concentration (Figure 5C). Interestingly, NaCl treatment did not decrease the K^+^ content in the pollen; rather, the K^+^ content increased in the NaCl-treated plants compared to the control, which maintains the Na^+^/K^+^ ratio at around 1 (Figure 5H). These findings are consistent with the K^+^ accumulation in the cytoplasm and nucleus of salt gland cells of the recretohalophyte *Limonium bicolor* observed using NanoSIMS (Feng et al., 2015). These results indicate that a certain amount of K^+^ and Na^+^ accumulation in reproductive organs is essential for the reproductive development of *S. salsa* under saline conditions.

Potassium is known to play an important role in reproductive growth by regulating carbohydrate and protein metabolism. In cotton (*Gossypium hirsutum*), for example, K^+^ deficiency results in a lower efficiency of seed set due to reduced carbohydrate and ATP contents in the K-deficient pistils (Hu et al., 2018). K^+^ contributes to pollen germination and tube growth (Fan et al., 2001) through its pivotal role in turgor pressure regulation (Rehman and Yun, 2006). We found that the K^+^ content in the vegetative organs of plants diminished with Na^+^ accumulation during NaCl treatment, but that it increased in the pollen grains (Figure 5D). This is in agreement with results from Guo et al. (2018) indicating that the higher seed set efficiency under NaCl treatment could be explained by an improvement in pollen tube growth in the style.

The high K^+^ content we observed in the reproductive organs of *S. salsa* treated with NaCl was accompanied by high expression levels of genes encoding K^+^ transporters, such as *SsCHX13*, *SsKEA3*, and *SsKEA5* (Figure 7). These upregulated genes may be associated with the high efficiency of K^+^ absorption and K^+^ homeostasis in *S. salsa* reproductive organs, such as pollen—much like the AtKEAs, which play a crucial role in K^+^ and pH homeostasis in Arabidopsis (Zheng et al., 2013; Kunz et al., 2014). There are six AtKEAs in Arabidopsis, among which AtKEA1–AtKEA3 function in the plastid. AtKEA3 is targeted to the thylakoid membrane, and given the increased pH seen in the *kea3* mutant, it likely contributes to chloroplast functions, such as osmoregulation and ion and pH homeostasis (Kunz et al., 2014; Sze and Chanroj, 2018), and responds to photosynthesis (Armbruster et al., 2016). In the case of *AtKEA5*, expression is induced by NaCl and ABA treatments as compared to that in untreated controls (Zheng et al., 2013). However, the localization of KEA5 in plants remains unknown (Chen et al., 2015). *SsAKT1*, encoding an inward potassium channel, was expressed at high levels in the flowers of *S. salsa* plants treated with NaCl in our study (Figure 7), as did the homolog in Arabidopsis, whose protein product has been confirmed to conduct K^+^ uptake under low K^+^ conditions (Wang et al., 2016). In addition, the gene expression pattern of K^+^ transporters during reproductive growth is quite different from that during vegetative growth (Shao et al., 2014). It is possible that *S. salsa* maintains a desirable Na^+/^K^+^ ratio in the reproductive organs by greatly upregulating the expression of the related genes, as *Phragmites karka* does in a saline environment (Abideen et al., 2014). Furthermore, another mechanism that increases flower number and improves anther development in *S. salsa* plants grown in a saline environment involves maintaining a certain K^+^/Na^+^ ratio and a certain concentration of Na^+^ in the reproductive cells.

Sodium is beneficial to certain C_4_ plant species at low concentrations, but in the case of euhalophytes, the beneficial levels are far higher (Flowers and Colmer, 2008). It has been reported that Na^+^ in growth medium at a concentration of several millimolar can replace K^+^ and have a positive effect on plant growth and yield (Kronzucker et al., 2013). Halophyte growth has been found to be stimulated, both in the seed germination period (Deng et al., 2014) and the vegetative growth period (Wang et al., 2012), as well as in the reproductive growth period (Guo et al., 2018), when NaCl is supplied in the growth medium at concentrations of 200 mM or higher.

In saline environments, the activity of carrier proteins, located in the plasma membrane or tonoplast, can be regulated to maintain ion homeostasis and promote salt tolerance. For example, increased expression of the plasma membrane and tonoplast Na^+^/H^+^ antiporter has been found to enhance salt tolerance in Arabidopsis (Yang et al., 2009), transgenic tobacco (*Nicotiana tabacum*; Yue et al., 2012), and tomato (Olías et al., 2009).

In the present study, the relative expression levels of the tonoplast K^+^| Na^+^/H^+^ antiporter gene *SsNHX1* were significantly increased under high salinity conditions (Figure 7), which could promote Na^+^ compartmentalization into vacuoles, thus reducing the cell osmotic potential and preventing ion toxicity. This could also lead to Na^+^ accumulation in the flowers (Guo et al., 2018) and pollen grains (Figure 5B) of *S. salsa* treated with NaCl. While the plasma membrane-localized Na^+^/H^+^ antiporter SOS1 plays an important role in extruding Na^+^ from the cytosol to the apoplast, upregulated expression of *SsSOS1* in flowers could maintain the intracellular Na^+^/K^+^ balance in reproductive organs of *S. salsa*. In saline environments, accumulated Na^+^ reduces the uptake of K^+^ and alters the Na^+^/K^+^ ratios in plant cells, thereby interrupting normal cellular metabolism. Therefore, like the K^+^ content (Anschütz et al., 2014), the Na^+^/K^+^ ratio in plant cells is a crucial parameter of plant salt tolerance and growth in saline environments (Chen et al., 2007; Hauser and Horie, 2010). We found that *S. salsa* plants grown under high-salt conditions, compared with control plants grown under low-salt conditions, maintained a higher level of K^+^ in pollen grains (Figure 5D) and a lower Na^+^/K^+^ ratio in pollen grains (Figure 5H), and their reproductive growth was enhanced (Guo et al., 2018). The higher relative K^+^ content and lower Na^+^/K^+^ ratio in the reproductive organs contributed to the improved reproduction of *S. salsa* in saline conditions compared with control conditions. Furthermore, maintaining a low Na^+^ and high K^+^ concentration in the flowers is mediated by the high relative expression of K^+^ and Na^+^ transporter genes, such as *SsCHX13* and *SsCHX14*, when *S. salsa* plants are exposed to 200 mM NaCl (Figure 7). This was consistent with the pattern of *OsCHX14* expression in rice (*Oryza sativa*), which is preferentially expressed in flowers and participates in ion homeostasis in the flowers (Chen et al., 2016). In Arabidopsis, CHX14 is localized in the plasma membrane, along with CHX13, and both proteins contribute to the redistribution of K^+^ (Zhao et al., 2015).

Cl^−^ is an essential plant micronutrient (Hänsch and Mendel, 2009). A high Cl^−^ content was found in both vegetative organs and reproductive organs of the halophyte *S. salsa*. Furthermore, the relative expression level of *SsCLC* in *S. salsa* flowers was significantly increased by treatment with NaCl. The chloride channel (CLC) is essential for nutrition, stress resistance, and ion homeostasis, and the results of the present study are consistent with data from Arabidopsis indicating that CLC is required for anion homeostasis and pH adjustment (Jossier et al., 2010). Seven *CLC* genes exist in the Arabidopsis genome, and their protein products have been shown to participate in Cl^−^ transport across the tonoplast and to mediate plant salinity tolerance (Jossier et al., 2010). An appropriate concentration of Na^+^ and Cl^−^, and an appropriate K^+^/Na^+^ ratio in the reproductive cells contributed to flower and seed development of *S. salsa* treated with NaCl. For the euhalophyte *S. salsa*, growth in a non-saline environment may constitute a stress (Cheeseman, 2015).

The processes of ion homeostasis, including ion and proton transport, require the PMF, which is generated by pump proteins in the plasma membrane (H^+^-ATPases) and tonoplast (V-ATPases), as well as H^+^-pyrophosphatases such as AVP1 (Hasegawa, 2013). Observations with intracellular microelectrodes have shown that electrogenic H^+^ pumping creates a transient hyperpolarization (−250 mV) and tends to depolarize the membrane potential to −155 mV, thus forming the PMF and providing electrical energy for ion transport (Higinbotham, 1973). Besides its normal function in the plant cell, H^+^ pumping also plays critical roles in salt resistance: in one study, for example, the salt tolerance of Arabidopsis was improved by overexpression of a vacuolar H^+^-ATPase subunit E1 gene (*LmVHA-E1*) from the halophyte *Lobularia maritima* (Dabbous et al., 2017). Zhang et al. (2017) also showed that the upregulation of plasma membrane H^+^-ATPase activity could enhance salt tolerance in Arabidopsis. There are two types of H^+^pyrophosphatases (H^+^-PPases) in Arabidopsis, types I and II, and the type II enzymes (AtVHP2s) are abundant in flowers (Segami et al., 2010). Our analysis of the relative expression levels of H^+^-ATPase (in the plasma membrane and tonoplast) and H^+^-pyrophosphatase genes revealed a significant increase in their relative expression levels in flowers from NaCl-treated versus control plants (Figure 7), especially for *SsAHA4* and *SsAVP1*. This is consistent with the role of the AHA4 isoform in salt tolerance (Rodrigues et al., 2014). The higher expression of these genes may provide a greater PMF for ion transport, and thereby improve the growth and resistance of *S. salsa* when treated with NaCl.

## Conclusion

**FIGURE 8:**
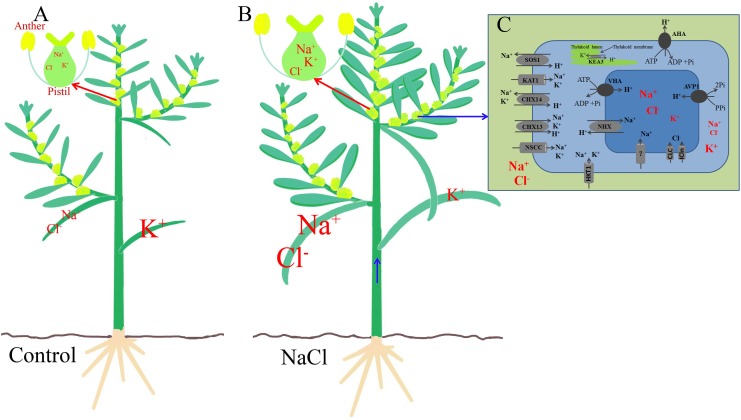
The possible mechanism of Na^+^, Cl^−^, and K^+^ homeostasis in the reproductive organs of the euhalophyte *S. salsa* under saline conditions. **(A)** The control condition (0 mM NaCl). **(B)** Saline treatment (200 mM NaCl). **(C)** Flower cell from a NaCl-treated plant. In *S. salsa* exposed to NaCl, ionic homeostasis in the flower cells is mainly dependent on Na^+^ compartmentalization into the vacuole by the Na^+^/H^+^ transporter in the tonoplast (NHX) and Na^+^ extrusion to the apoplast by the Na^+^/H^+^ transporter in the plasma membrane (SOS1), as well as on K^+^ transport, by proteins such as KEA and AKT1, and Cl^−^ transport through CLC and ICln. The driving force is provided by proton pumps in the plasma membrane and tonoplast (e.g., AHA, VHA, and AVP1). The sizes of the symbols for ions such as Na^+^, Cl^−^, and K^+^ and transporters such as AKT1 represent their relative contents in the different organs or cells.

A 200 mM NaCl treatment significantly increased the Na^+^ and Cl^−^ contents in both the vegetative and reproductive organs of *S. salsa*, but promoted the reproductive growth process, and the seed number increased mainly via increases in the differentiation of flower buds, the development of anthers, and the number of brown seeds. In a saline environment, Na^+^ enters *S. salsa* cells mainly through the high-affinity K^+^ transporter HKT1 and non-selective cation channels NSCC on the plasma membrane, and excess Na^+^ is compartmentalized into the vacuole through the intracellular Na^+^/H^+^ antiporter NHX1 on the tonoplast, thereby maintaining a relatively higher Na^+^ content in the leaves, stems, and floral organs than in control plants not exposed to high levels of NaCl. The ionic compartmentalization into the vacuole has two roles. First, it reduces the Na^+^ content in the cytoplasm and provides detoxification. Second, it performs osmotic adjustment. During upward transport in the xylem, Na^+^ is unloaded from xylem vessels by HKT1 and the Na^+^ content in the xylem vessels gradually decreases. Thus, compared with the Na^+^ content in the vegetative organs (leaves and stems), that in the reproductive organs was lower, but still higher than that in the control plants. The NaCl treatment did not decrease the K^+^ content in the pistils significantly, and it increased the K^+^ content in pollen grains. The higher K^+^ content in the reproductive cells may have resulted from specific higher expression of K^+^ transporter genes (such as *SsAKT1*, *SsKEA3*, and *SsKEA5*) in the flowers of NaCl-treated plants. The ionic homeostasis and the high K^+^/Na^+^ ratio in the reproductive organs and pollen of *S. salsa* treated with NaCl comprise a specific mechanism that enables *S. salsa* to maintain better growth conditions for reproduction than it could without the extra NaCl. Ion homeostasis could have been achieved by the increased expression in the flowers of genes, such as *SsSOS1*, *SsAKT1*, *SsKEA3*, *SsCLC*, *SsNHX1*, *SsCHX13*, and *SsCHX14*, as well as the proton pump genes, such as *SsAHA4*, *SsVHA*, and *SsAVP1*. We propose a possible pathway whereby the reproductive organs of *S. salsa* distribute Na^+^ and Cl^−^ between leaves, stems, and flowers (Figure 8). In saline conditions, the improved reproductive growth of the halophyte was accompanied by ionic homeostasis in reproductive cells, and the upregulation of specific genes that function in flowers. The experimental evidence presented here provides insights into the regulation of the salinity stress response during the reproductive growth of halophytes.

## Author Contributions

JG and BW conceived the original project, designed the experiments, and wrote the article. JG and XD performed most of the experiments. XD and GH performed the statistical analysis. All authors read and approved the final manuscript.

## Conflict of Interest Statement

The authors declare that the research was conducted in the absence of any commercial or financial relationships that could be construed as a potential conflict of interest.

## Supplementary Material

FIGURE S1 Growth parameters of *Suaeda salsa* plants. Plant fresh weight **(A)**, dry weight **(B)**, plant height **(C)**, and the first branch number **(D)** of *S. salsa* plants grown in sand with a control nutrient solution and 200 mM NaCl treatment at the end of vegetative growth stages, respectively. Values are presented as the means ± SD of six replicates. Different letters indicate a significant difference at *P* ≤ 0.05 according to Duncan’s test.

FIGURE S2 Functional annotation of assembled sequences based on gene ontology (GO) categorization. The results are summarized for two main Go categories: Biological Process and Molecular Function.

TABLE S1 Data quality assessment of RNA-seq of *S. salsa* flowers unigenes.

TABLE S2 Length distribution of transcripts and unigenes in the *S. salsa* flower reference transcriptome.

TABLE S3 Assembly statistics for the *S. salsa* flower reference transcriptome.

TABLE S4 Gene function annotations in seven databases, including: NR, NT, KO, Swiss-prot, PFAM, GO, and KOG/COG, the gene number and the percentage of the total genes.

TABLE S5 Primer pairs for quantitative real-time PCR.

TABLE S6 List of all identified DEGs in *S. salsa* flowers between control and NaCl-treated groups.

## References

[B1] Abideen Z. Koyro H. W. Huchzermeyer B. Ahmed M. Z. Gul B. Khan M. A. (2014). Moderate salinity stimulates growth and photosynthesis of *Phragmites karka* by water relations and tissue specific ion regulation. *Environ. Exp. Bot.* 105 70–76. 10.1016/j.envexpbot.2014.04.009

[B2] Amzallag G. N. (2005). Perturbed reproductive development in salt treated *Sorghum bicolor*: a consequence of modifications in regulation network? *J. Exp. Bot.* 56 2821–2829. 10.1093/jxb/eri274 16143716

[B3] Anschütz U. Becker D. Shabala S. (2014). Going beyond nutrition: regulation of potassium homoeostasis as a common denominator of plant adaptive responses to environment. *J. Plant Physiol.* 171 670–687. 10.1016/j.jplph.2014.01.009 24635902

[B4] Apweiler R. Bairoch A. Wu C. H. Barker W. C. Boeckmann B. Ferro S. (2004). UniProt: the universal protein knowledgebase. *Nucleic Acids Res.* 32 D115–D119. 10.1093/nar/gkh131 14681372 PMC308865

[B5] Armbruster U. Leonelli L. Correa Galvis V. Strand D. Quinn E. H. Jonikas M. C. (2016). Regulation and levels of the thylakoid K+/H+ antiporter KEA3 shape the dynamic response of photosynthesis in fluctuating light. *Plant Cell Physiol.* 57 1557–1567. 10.1093/pcp/pcw085 27335350 PMC4937787

[B6] Ashburner M. Ball C. A. Blake J. A. Botstein D. Butler H. Cherry J. M. (2000). Gene ontology: tool for the unification of biology. *Nat. Genet.* 25 25–29. 10.1038/75556 10802651 PMC3037419

[B7] Baby T. Collins C. Tyerman S. D. Gilliham M. (2016). Salinity negatively affects pollen tube growth and fruit set in grapevines and cannot be ameliorated by silicon. *Am. J. Enol. Viticult.* 67 218–228. 10.5344/ajev.2015.15004

[B8] Barragán V. Leidi E. O. Andrés Z. Rubio L. De Luca A. Fernández J. A. (2012). Ion exchangers NHX1 and NHX2 mediate active potassium uptake into vacuoles to regulate cell turgor and stomatal function in *Arabidopsis*. *Plant Cell* 24 1127–1142. 10.1105/tpc.111.095273 22438021 PMC3336136

[B9] Bassil E. Tajima H. Liang Y. C. Ohto M. A. Ushijima K. Nakano R. (2011). The *Arabidopsis* Na+/H+ antiporters NHX1 and NHX2 control vacuolar pH and K+ homeostasis to regulate growth, flower development, and reproduction. *Plant Cell* 23 3482–3497. 10.1105/tpc.111.089581 21954467 PMC3203450

[B10] Cheeseman J. M. (2015). The evolution of halophytes, glycophytes and crops, and its implications for food security under saline conditions. *New Phytol.* 206 557–570. 10.1111/nph.13217 25495078

[B11] Chen L. Ren J. Shi H. Zhang Y. You Y. Fan J. (2015). *TdCBL6*, a calcineurin B-like gene from wild emmer wheat (*Triticum dicoccoides*), is involved in response to salt and low-K+ stresses. *Mol. Breed.* 35:50 10.1007/s11032-015-0229-1

[B12] Chen M. Yang Z. Liu J. Zhu T. T. Wei X. C. Fan H. (2018). Adaptation mechanism of salt excluders under saline conditions and its applications. *Int. J. Mol. Sci.* 19:3668. 10.3390/ijms19113668 30463331 PMC6274768

[B13] Chen Y. Ma J. Miller A. J. Luo B. Wang M. Zhu Z. (2016). OsCHX14 is involved in the K+ homeostasis in rice (*Oryza sativa*) flowers. *Plant Cell Physiol.* 57 1530–1543. 10.1093/pcp/pcw088 27903806

[B14] Chen Z. Pottosin I. I. Cuin T. A. Fuglsang A. T. Tester M. Jha D. (2007). Root plasma membrane transporters controlling K+/Na+ homeostasis in salt-stressed barley. *Plant Physiol.* 145 1714–1725. 10.1104/pp.107.110262 17965172 PMC2151677

[B15] Cuartero J. Fernández-Muñoz R. (1998). Tomato and salinity. *Sci. Hortic.* 78 83–125. 10.1016/S0304-4238(98)00191-5

[B16] Dabbous A. Saad R. B. Brini F. Farhat-Khemekhem A. Zorrig W. Abdely C. (2017). Over-expression of a subunit E1 of a vacuolar H+-ATPase gene (*LmVHA-E1*) cloned from the halophyte *Lobularia maritima* improves the tolerance of *Arabidopsis thaliana* to salt and osmotic stresses. *Environ. Exp. Bot.* 137 128–141. 10.1016/j.envexpbot.2017.01.013

[B17] Davidson N. M. Oshlack A. (2014). Corset: enabling differential gene expression analysis for de novo assembled transcriptomes. *Genome Biol.* 15:410. 10.1186/s13059-014-0410-6 25063469 PMC4165373

[B18] Deinlein U. Stephan A. B. Horie T. Luo W. Xu G. Schroeder J. I. (2014). Plant salt-tolerance mechanisms. *Trends Plant Sci.* 19 371–379. 10.1016/j.tplants.2014.02.001 24630845 PMC4041829

[B19] Deng M. H. Wen J. F. Huo J. L. Zhu H. S. Dai X. Z. Zhang Z. Q. (2012). Relationship of metabolism of reactive oxygen species with cytoplasmic male sterility in pepper (*Capsicum annuum L.*). *Sci. Hortic.* 134 232–236. 10.1016/j.scienta.2011.10.027

[B20] Deng Y. Yuan F. Feng Z. Ding T. Song J. Wang B. (2014). Comparative study on seed germination characteristics of two species of Australia saltbush under salt stress. *Acta Ecol. Sin.* 34 337–341. 10.1016/j.chnaes.2013.07.011

[B21] Deng Y. Q. Bao J. Yuan F. Liang X. Feng Z. T. Wang B. S. (2016). Exogenous hydrogen sulfide alleviates salt stress in wheat seedlings by decreasing Na+ content. *Plant Growth Regul.* 79 391–399. 10.1007/s10725-015-0143-x

[B22] Durand M. Lacan D. (1994). Sodium partitioning within the shoot of soybean. *Physiol. Plantarum* 91 65–71. 10.1111/j.1399-3054.1994.tb00660.x

[B23] Endo M. Tsuchiya T. Hamada K. Kawamura S. Yano K. Ohshima M. (2009). High temperatures cause male sterility in rice plants with transcriptional alterations during pollen development. *Plant Cell Physiol.* 50 1911–1922. 10.1093/pcp/pcp135 19808807

[B24] Fan L. M. Wang Y. F. Wang H. Wu W. H. (2001). In vitro *Arabidopsis* pollen germination and characterization of the inward potassium currents in *Arabidopsis* pollen grain protoplasts. *J. Exp. Bot.* 52 1603–1614. 10.1093/jexbot/52.361.1603 11479325

[B25] Feng Z. T. Deng Y. Q. Zhang S. C. Liang X. Yuan F. Hao J. L. (2015). K+ accumulation in the cytoplasm and nucleus of the salt gland cells of *Limonium bicolor* accompanies increased rates of salt secretion under NaCl treatment using NanoSIMS. *Plant Sci.* 238 286–296. 10.1016/j.plantsci.2015.06.021 26259195

[B26] Flowers T. J. Colmer T. D. (2008). Salinity tolerance in halophytes. *New Phytol.* 179 945–963. 10.1111/j.1469-8137.2008.02531.x 18565144

[B27] Flowers T. J. Troke P. F. Yeo A. R. (1977). The mechanism of salt tolerance in halophytes. *Annu. Rev. Plant Biol.* 28 89–121. 10.1146/annurev.pp.28.060177.000513

[B28] Flowers T. J. Yeo A. R. (1986). Ion relations of plants under drought and salinity. *Aust. J. Plant Physiol.* 13 75–91. 10.1071/PP9860075

[B29] Forieri I. Hildebrandt U. Rostás M. (2016). Salinity stress effects on direct and indirect defence metabolites in maize. *Environ. Exp. Bot.* 122 68–77. 10.1016/j.envexpbot.2015.09.007

[B30] Grabherr M. G. Haas B. J. Yassour M. Levin J. Z. Thompson D. A. Amit I. (2011). Full-length transcriptome assembly from RNA-Seq data without a reference genome. *Nat. Biotechnol.* 29 644–652. 10.1038/nbt.1883 21572440 PMC3571712

[B31] Greenway H. Munns R. (1980). Mechanisms of salt tolerance in nonhalophytes. *Annu. Rev. Plant Physiol.* 31 149–190. 10.1146/annurev.pp.31.060180.001053

[B32] Grigore M. N. Boscaiu M. Llinares J. Vicente O. (2012). Mitigation of salt stress-induced inhibition of Plantago crassifolia reproductive development by supplemental calcium or magnesium. *Not. Bot. Hortic. Agrobo.* 40 58–66. 10.15835/nbha4028246

[B33] Grunberg K. Fernández-Muñoz R. Cuartero J. (1995). Growth, flowering, and quality and quantity of pollen of tomato plants grown under saline conditions. *Acta Hortic.* 412 484–489. 10.17660/ActaHortic.1995.412.58

[B34] Guo J. Li Y. Han G. Song J. Wang B. (2018). NaCl markedly improved the reproductive capacity of the euhalophyte *Suaeda salsa*. *Funct. Plant Biol.* 45 350–361. 10.1071/FP17181 32290958

[B35] Guo J. Suo S. Wang B. S. (2015). Sodium chloride improves seed vigour of the euhalophyte *Suaeda salsa*. *Seed Sci. Res.* 25 335–344. 10.1017/S0960258515000239

[B36] Hamamoto S. Horie T. Hauser F. Deinlein U. Schroeder J. I. Uozumi N. (2015). HKT transporters mediate salt stress resistance in plants: from structure and function to the field. *Curr. Opin. Biotech.* 32 113–120. 10.1016/j.copbio.2014.11.025 25528276

[B37] Han N. Shao Q. Lu C. M. Wang B. S. (2005). The leaf tonoplast V-H+-ATPase activity of a C3 halophyte *Suaeda salsa* is enhanced by salt stress in a Ca-dependent mode. *J. Plant Physiol.* 162 267–274. 10.1016/j.jplph.2004.07.016 15832678

[B38] Hänsch R. Mendel R. R. (2009). Physiological functions of mineral micronutrients (Cu, Zn, Mn, Fe, Ni, Mo, B, Cl). *Curr. Opin. Plant Biol.* 12 259–266. 10.1016/j.pbi.2009.05.006 19524482

[B39] Hasegawa P. M. (2013). Sodium (Na+) homeostasis and salt tolerance of plants. *Environ. Exp. Bot.* 92 19–31. 10.1016/j.envexpbot.2013.03.001

[B40] Hauser F. Horie T. (2010). A conserved primary salt tolerance mechanism mediated by HKT transporters: a mechanism for sodium exclusion and maintenance of high K+/Na+ ratio in leaves during salinity stress. *Plant Cell Environ.* 33 552–565. 10.1111/j.1365-3040.2009.02056.x 19895406

[B41] Higinbotham N. (1973). Electropotentials of plant cells. *Annu. Rev. Plant Physiol.* 24 25–46. 10.1146/annurev.pp.24.060173.000325

[B42] Hu W. Loka D. A. Fitzsimons T. R. Zhou Z. Oosterhuis D. M. (2018). Potassium deficiency limits reproductive success by altering carbohydrate and protein balances in cotton (*Gossypium hirsutum L.*). *Environ. Exp. Bot.* 145 87–94. 10.1016/j.envexpbot.2017.10.024

[B43] Jarvis D. E. Ho Y. S. Lightfoot D. J. Schmöckel S. M. Li B. Borm T. J. (2017). The genome of chenopodium quinoa. *Nature* 542 307–312. 10.1038/nature21370 28178233

[B44] Jossier M. Kroniewicz L. Dalmas F. Thiec D. L. Ephritikhine G. Thomine S. (2010). The *Arabidopsis* vacuolar anion transporter, *AtCLCc*, is involved in the regulation of stomatal movements and contributes to salt tolerance. *Plant J.* 64 563–576. 10.1111/j.1365-313X.2010.04352.x 20822503

[B45] Kanehisa M. Araki M. Goto S. Hattori M. Hirakawa M. Itoh M. (2008). KEGG for linking genomes to life and the environment. *Nucleic Acids Res.* 36 D480–D484. 18077471 10.1093/nar/gkm882 PMC2238879

[B46] Khan M. A. Abdullah Z. (2003). Salinity–sodicity induced changes in reproductive physiology of rice (*Oryza sativa*) under dense soil conditions. *Environ. Exp. Bot.* 49 145–157. 10.1016/S0098-8472(02)00066-7

[B47] Khatun S. Flowers T. J. (1995). Effects of salinity on seed set in rice. *Plant Cell Environ.* 18 61–67. 10.1111/j.1365-3040.1995.tb00544.x

[B48] Khatun S. Rizzo C. A. Flowers T. J. (1995). Genotypic variation in the effect of salinity on fertility in rice. *Plant Soil* 173 239–250. 10.1007/BF00011461

[B49] Kotula L. Khan H. A. Quealy J. Turner N. C. Vadez V. Siddique K. H. M. (2015). Salt sensitivity in chickpea (*Cicer arietinum L.*): ions in reproductive tissues and yield components in contrasting genotypes. *Plant Cell Environ.* 38 1565–1577. 10.1111/pce.12506 25615287

[B50] Kronzucker H. J. Coskun D. Schulze L. M. Wong J. R. Britto D. T. (2013). Sodium as nutrient and toxicant. *Plant Soil* 369 1–23. 10.1007/s11104-013-1801-2

[B51] Kunz H. H. Gierth M. Herdean A. Satoh-Cruz M. Kramer D. M. Spetea C. (2014). Plastidial transporters KEA1, -2, and-3 are essential for chloroplast osmoregulation, integrity, and pH regulation in *Arabidopsis*. *Proc. Natl Acad. Sci. U. S. A.* 111 7480–7485. 10.1073/pnas.1323899111 24794527 PMC4034250

[B52] Leidi E. O. Barragán V. Rubio L. El-Hamdaoui A. Ruiz M. T. Cubero B. (2010). The AtNHX1 exchanger mediates potassium compartmentation in vacuoles of transgenic tomato. *Plant J.* 61 495–506. 10.1111/j.1365-313X.2009.04073.x 19912566

[B53] Li B. Dewey C. N. (2011). RESM: accurate transcript quantification from RNA-Seq data with or without a reference genome. *BMC Bioinformatics* 12:323. 10.1186/1471-2105-12-323 21816040 PMC3163565

[B54] Li K. Wang Y. Han C. Zhang W. Jia H. Li X. (2007). GA signaling and CO/FT regulatory module mediate salt-induced late flowering in *Arabidopsis thaliana*. *Plant Growth Regu.* 53 195–206. 10.1007/s10725-007-9218-7

[B55] Li X. Zhang X. Song J. Fan H. Feng G. Wang B. (2011). Accumulation of ions during seed development under controlled saline conditions of two *Suaeda salsa* populations is related to their adaptation to saline environments. *Plant soil* 341 99–107. 10.1007/s11104-010-0625-6

[B56] Livak K. J. Schmittgen T. D. (2001). Analysis of relative gene expression data using real-time quantitative PCR and the 2(-ΔΔCT) method. *Methods* 25 402–408. 10.1006/meth.2001.1262 11846609

[B57] Loka D. A. Oosterhuis D. M. (2014). Water-deficit stress effects on pistil biochemistry and leaf physiology in cotton (*Gossypium hirsutum L.*). *S. Afr. J. Bot.* 93 131–136. 10.1016/j.sajb.2014.03.019

[B58] Love M. I. Huber W. Anders S. (2014). Moderated estimation of fold change and dispersion for RNA-seq data with DESeq2. *Genome Biol.* 15:550. 10.1186/s13059-014-0550-8 25516281 PMC4302049

[B59] Lu C. Qiu N. Wang B. Zhang J. (2003). Salinity treatment shows no effects on photosystem II photochemistry, but increases the resistance of photosystem II to heat stress in halophyte *Suaeda salsa*. *J. Exp. Bot.* 54 851–860. 10.1093/jxb/erg080 12554728

[B60] Ma Q. Zhou X. R. Wu G. Q. Wang S. M. (2009). Cloning and sequence analysis of Actin gene gragment from the halophyte *Suaeda glauca*. *Biotechnology* 19 1–3.

[B61] Munns R. (2002). Comparative physiology of salt and water stress. *Plant Cell Environ.* 25 239–250. 10.1046/j.0016-8025.2001.00808.x 11841667

[B62] Munns R. Tester M. (2008). Mechanisms of salinity tolerance. *Annu. Rev. Plant Biol.* 59 651–681. 10.1146/annurev.arplant.59.032607.092911 18444910

[B63] Nguyen G. N. Hailstones D. L. Wilkes M. Sutton B. G. (2010). Drought stress: Role of carbohydrate metabolism in drought-induced male sterility in rice anthers. *J. Agron. crop Sci.* 196 346–357. 10.1111/j.1439-037X.2010.00423.x

[B64] Noctor G. (2015). Keeping a cool head: gene networks underlying chilling-induced male sterility in rice. *Plant Cell Environ.* 38 1252–1254. 10.1111/pce.12513 25651873

[B65] Olías R. Eljakaoui Z. Pardo J. M. Belver A. (2009). The Na+/H+ exchanger SOS1 controls extrusion and distribution of Na+ in tomato plants under salinity conditions. *Plant Signal. Behav.* 4 973–976. 10.4161/psb.4.10.9679 19826225 PMC2801365

[B66] Oliver S. N. Dennis E. S. Dolferus R. (2007). ABA regulates apoplastic sugar transport and is a potential signal for cold-induced pollen sterility in rice. *Plant Cell Physiol.* 48 1319–1330. 10.1093/pcp/pcm100 17693452

[B67] Oliver S. N. Van Dongen J. T. Alfred S. C. Mamun E. A. Zhao X. Saini H. S. (2005). Cold-induced repression of the rice anther-specific cell wall invertase gene *OSINV4* is correlated with sucrose accumulation and pollen sterility. *Plant Cell Environ.* 28 1534–1551. 10.1111/j.1365-3040.2005.01390.x

[B68] Onyemaobi I. Liu H. Siddique K. H. Yan G. (2017). Both male and female malfunction contributes to yield reduction under water stress during meiosis in bread wheat. *Front. Plant Sci.* 7:2071. 10.3389/fpls.2016.02071 28119733 PMC5222847

[B69] Pacini E. Dolferus R. (2016). “The trials and tribulations of the plant male gametophyte-understanding reproductive stage stress tolerance,” in *Abiotic and Biotic Stress in Plants-Recent Advances and Future Perspectives*, ed. Shanker A. (Rijeka: InTech), 703–754.

[B70] Pang C. H. Zhang S. J. Gong Z. Z. Wang B. S. (2005). NaCl treatment markedly enhances H2O2-scavenging system in leaves of halophyte *Suaeda salsa L*. *Physiol. Plantarum* 124 490–499.

[B71] Parvin K. Ahamed K. U. Islam M. M. Haque M. N. Hore P. K. Siddik M. A. (2015). Reproductive behavior of tomato plant under saline condition with exogenous application of calcium. *Middle East J. Sci. Res.* 23 2920–2926.

[B72] Plackett A. R. Thomas S. G. Wilson Z. A. Hedden P. (2011). Gibberellin control of stamen development: a fertile field. *Trends Plant Sci.* 16 568–578. 10.1016/j.tplants.2011.06.007 21824801

[B73] Pruitt K. D. Tatusova T. Maglott D. R. (2005). NCBI Reference Sequence (RefSeq): a curated non-redundant sequence database of genomes, transcripts and proteins. *Nucleic Acids Res.* 33 D501–D504. 10.1093/nar/gki025 15608248 PMC539979

[B74] Qiu N. Chen M. Guo J. Bao H. Ma X. Wang B. (2007). Coordinate up-regulation of V-H+-ATPase and vacuolar Na+/H+ antiporter as a response to NaCl treatment in a C3 halophyte *Suaeda salsa*. *Plant Sci.* 172 1218–1225. 10.1016/j.plantsci.2007.02.013

[B75] Rehman S. Yun S. (2006). Developmental regulation of K accumulation in pollen, anthers, and papillae: are anther dehiscence, papillae hydration, and pollen swelling leading to pollination and fertilization in barley (*Hordeum vulgare L.*) regulated by changes in K concentration? *J. Exp. Bot.* 57 1315–1321. 10.1093/jxb/erj106 16531463

[B76] Rengasamy P. (2006). World salinization with emphasis on Australia. *J. Exp. Bot.* 57 1017–1023. 10.1093/jxb/erj108 16510516

[B77] Rodrigues R. B. Sabat G. Minkoff B. B. Burch H. L. Nguyen T. T. Sussman M. R. (2014). Expression of a translationally fused TAP-tagged plasma membrane proton pump in *Arabidopsis thaliana*. *Biochemistry* 53 566–578. 10.1021/bi401096m 24397334 PMC3985734

[B78] Sahoo D. P. Kumar S. Mishra S. Kobayashi Y. Panda S. K. Sahoo L. (2016). Enhanced salinity tolerance in transgenic mungbean overexpressing *Arabidopsis* antiporter (NHX1) gene. *Mol. Breed.* 36:144 10.1007/s11032-016-0564-x

[B79] Samineni S. Siddique K. H. Gaur P. M. Colmer T. D. (2011). Salt sensitivity of the vegetative and reproductive stages in chickpea (*Cicer arietinum L.*): podding is a particularly sensitive stage. *Environ. Exp. Bot.* 71 260–268. 10.1016/j.envexpbot.2010.12.014

[B80] Segami S. Nakanishi Y. Sato M. H. Maeshima M. (2010). Quantification, organ-specific accumulation and intracellular localization of type II H+-pyrophosphatase in *Arabidopsis thaliana*. *Plant Cell Physiol.* 51 1350–1360. 10.1093/pcp/pcq096 20605924

[B81] Shao Q. Han N. Ding T. Zhou F. Wang B. (2014). SsHKT1;1 is a potassium transporter of the C3 halophyte *Suaeda salsa* that is involved in salt tolerance. *Funct. Plant Biol.* 41 790–802. 10.1071/FP13265 32481033

[B82] Sheoran I. S. Saini H. S. (1996). Drought-induced male sterility in rice: changes in carbohydrate levels and enzyme activities associated with the inhibition of starch accumulation in pollen. *Sex. Plant Reprod.* 9 161–169. 10.1007/BF02221396

[B83] Shi H. Lee B. H. Wu S. J. Zhu J. K. (2003). Overexpression of a plasma membrane Na+/H+ antiporter gene improves salt tolerance in *Arabidopsis thaliana*. *Nat. Biotechnol.* 21 81–85. 10.1038/nbt766 12469134

[B84] Slama I. Abdell C. Bouchereau A. Flowers T. Savoure A. (2015). Diversity, distribution and roles of osmopretective compounds accumulated in halophytes under abiotic stress. *Ann. Bot.* 115 433–437. 10.1093/aob/mcu239 25564467 PMC4332610

[B85] Sohrabi Y. Heidari G. Esmailpoor B. (2008). Effect of salinity on growth and yield of desi and kabuli chickpea cultivars. *Pakistan J. Biol. Sci.* 11 664–667. 10.3923/pjbs.2008.664.667 18817146

[B86] Song J. Chen M. Feng G. Jia Y. Wang B. Zhang F. (2009). Effect of salinity on growth, ion accumulation and the roles of ions in osmotic adjustment of two populations of *Suaeda salsa*. *Plant Soil* 314 133–141. 10.1007/s11104-008-9712-3

[B87] Song J. Fan H. Zhao Y. Jia Y. Du X. Wang B. (2008). Effect of salinity on germination, seedling emergence, seedling growth and ion accumulation of a euhalophyte *Suaeda salsa* in an intertidal zone and on saline inland. *Aquat. Bot.* 88 331–337. 10.1016/j.aquabot.2007.11.004

[B88] Song J. Shi G. Gao B. Fan H. Wang B. (2011). Waterlogging and salinity effects on two *Suaeda salsa* populations. *Physiol. Plantarum* 141 343–351. 10.1111/j.1399-3054.2011.01445.x 21214881

[B89] Song J. Wang B. (2015). Using euhalophytes to understand salt tolerance and to develop saline agriculture: *Suaeda salsa* as a promising model. *Ann. Bot.* 115 541–553. 10.1093/aob/mcu194 25288631 PMC4332605

[B90] Song J. Zhou J. Zhao W. Xu H. Wang F. Xu Y. (2016). Effects of salinity and nitrate on production and germination of dimorphic seeds applied both through the mother plant and exogenously during germination in *Suaeda salsa*. *Plant Spec. Biol.* 31 19–28. 10.1111/1442-1984.12071

[B91] Storme N. D. Geelen D. (2014). The impact of environmental stress on male reproductive development in plants: biological processes and molecular mechanisms. *Plant Cell Environ.* 37 1–18. 10.1111/pce.12142 23731015 PMC4280902

[B92] Sun K. Hunt K. Hauser B. A. (2004). Ovule abortion in *Arabidopsis* triggered by stress. *Plant Physiol.* 135 2358–2367. 10.1104/pp.104.043091 15299130 PMC520803

[B93] Sze H. Chanroj S. (2018). Plant endomembrane dynamics: studies of K+/H+ antiporters provide insights on the effects of pH and ion homeostasis. *Plant Physiol.* 177 875–895. 10.1104/pp.18.00142 29691301 PMC6053008

[B94] Tatusov R. L. Galperin M. Y. Natale D. A. Koonin E. V. (2000). The COG database: a tool for genome-scale analysis of protein functions and evolution. *Nucleic Acids Res.* 28 33–36. 10.1093/nar/28.1.33 10592175 PMC102395

[B95] Turner N. C. Colmer T. D. Quealy J. Pushpavalli R. Krishnamurthy L. Kaur J. (2013). Salinity tolerance and ion accumulation in chickpea (*Cicer arietinum L.*) subjected to salt stress. *Plant Soil* 365 347–361. 10.1007/s00709-015-0892-4 26468060

[B96] Vadez V. Krishnamurthy L. Serraj R. Gaur P. M. Upadhyaya H. D. Hoisington D. A. (2007). Large variation in salinity tolerance in chickpea is explained by differences in sensitivity at the reproductive stage. *Field Crops Res.* 104 123–129. 10.1016/j.fcr.2007.05.014

[B97] Vadez V. Rashmi M. Sindhu K. Muralidharan M. Pushpavalli R. Turner N. C. (2012). Large number of flowers and tertiary branches, and higher reproductive success increase yields under salt stress in chickpea. *Eur. J. Agron.* 41 42–51. 10.1016/j.eja.2012.03.008

[B98] Ventura Y. Myrzabayeva M. Alikulov Z. Omarov R. Khozin-Goldberg I. Sagi M. (2014). Effects of salinity on flowering, morphology, biomass accumulation and leaf metabolites in an edible halophyte. *AoB Plants* 6 55–64. 10.1093/aobpla/plu053 25178274 PMC4172196

[B99] Wang B. S. Luttge U. Ratajczak R. (2004). Specific regulation of SOD isoforms by NaCl and osmotic stress in leaves of C3 halophyte. *J. Plant Physiol.* 161 285–293. 10.1078/0176-1617-01123 15077627

[B100] Wang D. Wang H. Han B. Wang B. Guo A. Zheng D. (2012). Sodium instead of potassium and chloride is an important macronutrient to improve leaf succulence and shoot development for halophyte *Sesuvium portulacastrum*. *Plant Physiol. Biochem.* 51 53–62. 10.1016/j.plaphy.2011.10.009 22153240

[B101] Wang X. P. Chen L. M. Liu W. X. Shen L. K. Wang F. L. Zhou Y. (2016). AtKC1 and CIPK23 synergistically modulate AKT1-mediated low potassium stress responses in *Arabidopsis*. *Plant Physiol.* 170 2264–2277. 10.1104/pp.15.01493 26829980 PMC4825127

[B102] Yang M. F. Song J. Wang B. S. (2010). Organ-specific responses of vacuolar H+-ATPase in the shoots and roots of C3 halophyte *Suaeda salsa* to NaCl. *J. Integr. Plant Biol.* 52 308–314. 10.1111/j.1744-7909.2010.00895.x 20377691

[B103] Yang Q. Chen Z. Z. Zhou X. F. Yin H. B. Li X. Xin X. F. (2009). Overexpression of SOS (*Salt Overly Sensitive*) genes increases salt tolerance in transgenic *Arabidopsis*. *Mol. Plant* 2 22–31. 10.1093/mp/ssn058 19529826 PMC2639737

[B104] Yang Z. Wang Y. Wei X. C. Zhao X. Wang B. S. Sui N. (2017). Transcription profiles of genes related to hormonal regulations under salt stress in sweet sorghum. *Plant Mol. Biol. Rep.* 35 586–599. 10.1007/s11105-017-1047-x

[B105] Yue Y. Zhang M. Zhang J. Duan L. Li Z. (2012). *SOS1* gene overexpression increased salt tolerance in transgenic tobacco by maintaining a higher K+/Na+ ratio. *J. Plant Physiol.* 169 255–261. 10.1016/j.jplph.2011.10.007 22115741

[B106] Zhang Q. F. Li Y. Y. Pang C. H. Lu C. M. Wang B. S. (2005). NaCl stress enhances thylakoid-bound SOD activity in the leaves of C3 halophyte *Suaeda salsa L*. *Plant Sci.* 168 423–430. 10.1016/j.plantsci.2004.09.002

[B107] Zhang Y. Wang Y. Sa G. Zhang Y. Deng J. Deng S. (2017). *Populus euphratica* J3 mediates root K+/Na+ homeostasis by activating plasma membrane H+-ATPase in transgenic *Arabidopsis* under NaCl salinity. *Plant Cell Tiss. Org.* 131 75–88. 10.1007/s11240-017-1263-y

[B108] Zhao J. Li P. Motes C. M. Park S. Hirschi K. D. (2015). CHX14 is a plasma membrane K-efflux transporter that regulates K*+* redistribution in *Arabidopsis thaliana*. *Plant Cell Environ.* 38 2223–2238. 10.1111/pce.12524 25754420

[B109] Zhao K. F. Fan H. Ungar I. A. (2002). Survey of halophyte species in China. *Plant Sci.* 163 491–498. 10.1016/S0168-9452(02)00160-7

[B110] Zheng C. Dong J. Jian C. Dai T. (2010). Post-anthesis salinity and waterlogging and their combination affect uptake of potassium and sodium ions and starch accumulation in grain of wheat. *Acta Ecol. Sin.* 30 4756–4764.

[B111] Zheng S. Pan T. Fan L. Qiu Q. S. (2013). A novel AtKEA gene family, homolog of bacterial K+/H+ antiporters, plays potential roles in K+ homeostasis and osmotic adjustment in *Arabidopsis*. *PLoS One* 8:e81463. 10.1371/journal.pone.0081463 24278440 PMC3835744

