# Supplementary figures and images for: Salt-Enhanced Reproductive Development of Suaeda salsa L. Coincided With Ion Transporter Gene Upregulation in Flowers and Increased Pollen K+ Content
Source: Front Plant Sci. 2019 Mar 29;10:333. doi: 10.3389/fpls.2019.00333 (PMC6449877; doi:10.3389/fpls.2019.00333)

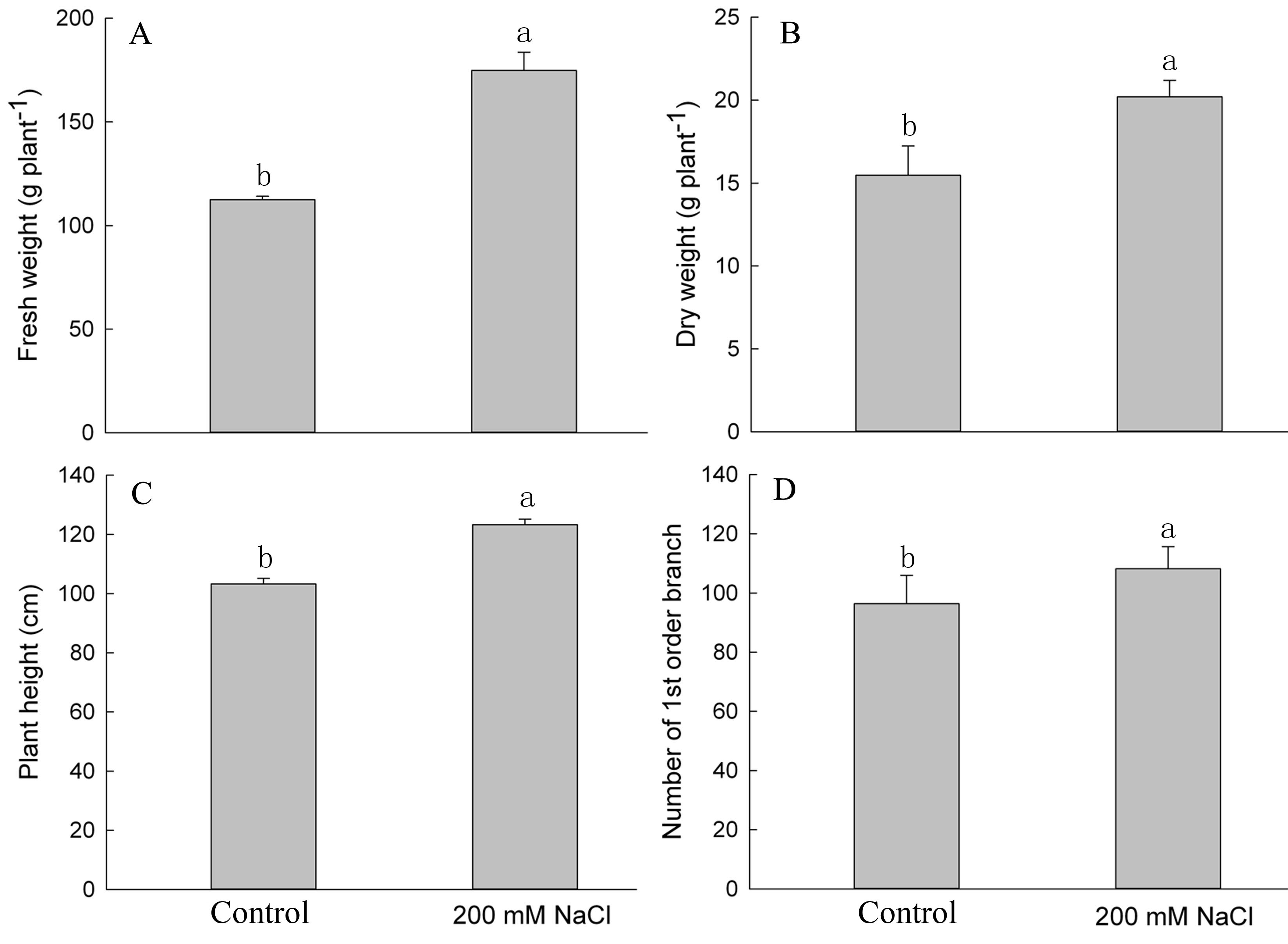

Supplement: FIGURE S1 — Growth parameters of Suaeda salsa plants. Plant fresh weight (A), dry weight (B), plant height (C), and the first branch number (D) of S. salsa plants grown in sand with a control nutrient solution and 200 mM NaCl treatment at the end of vegetative growth stages, respectively. Values are presented as the means ± SD of six replicates. Different letters indicate a significant difference at P ≤ 0.05 according to Duncan’s test. [file Image_1.JPEG]

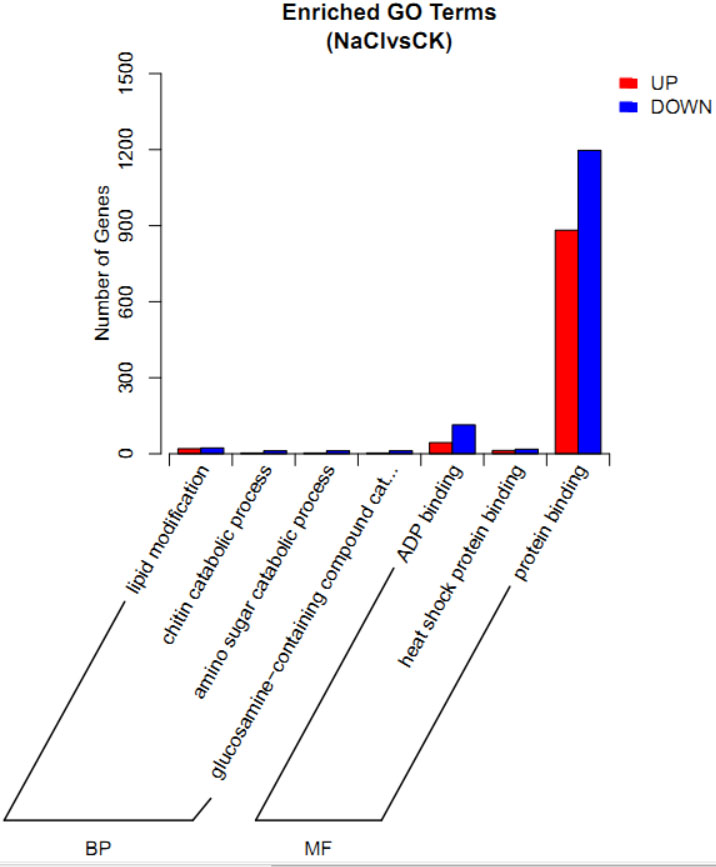

Supplement: FIGURE S2 — Functional annotation of assembled sequences based on gene ontology (GO) categorization. The results are summarized for two main Go categories: Biological Process and Molecular Function. [file Image_2.JPEG]
