# Supplementary material for: Salt-Enhanced Reproductive Development of Suaeda salsa L. Coincided With Ion Transporter Gene Upregulation in Flowers and Increased Pollen K+ Content
Source: Front Plant Sci. 2019 Mar 29;10:333. doi: 10.3389/fpls.2019.00333 (PMC6449877; doi:10.3389/fpls.2019.00333)
Supplement: TABLE S1 — Data quality assessment of RNA-seq of S. salsa flowers unigenes. [file Table_1.docx]

Table S1 Data quality assessment of RNA-seq of *S. salsa* flowers unigenes

| Sample | Raw reads | Clean reads | | Clean bases | Q20(%) | Q30(%) | GC(%) |
| --- | --- | --- | --- | --- | --- | --- | --- |
| CK_1 | 76611254 | 74425060 | 11.16G | | 96.30 | 94.60 | 42.29 |
| CK_2 | 72623358 | 69265038 | 10.39G | | 97.59 | 93.54 | 43.84 |
| CK_3 | 86281136 | 84490870 | 12.67G | | 95.88 | 93.83 | 42.16 |
| NaCl_1 | 68271536 | 65606518 | 9.84G | | 96.28 | 94.57 | 42.16 |
| NaCl_2 | 76611254 | 74019506 | 11.1G | | 95.70 | 93.58 | 42.06 |
| NaCl_3 | 67451472 | 65755280 | 9.86G | | 97.65 | 93.61 | 42.12 |

Sample: CK: samples of control; NaCl: samples of treated with 200 mM NaCl. Clean reads: The number of reads after removing low-quality sequences. The subsequent analysis is based on clean reads. Q20 and Q30, the percentage of bases with Phred values >20 and >30, respectively. GC content: the GC ratio of the total base number.
