# Supplementary material for: Salt-Enhanced Reproductive Development of Suaeda salsa L. Coincided With Ion Transporter Gene Upregulation in Flowers and Increased Pollen K+ Content
Source: Front Plant Sci. 2019 Mar 29;10:333. doi: 10.3389/fpls.2019.00333 (PMC6449877; doi:10.3389/fpls.2019.00333)
Supplement: TABLE S2 — Length distribution of transcripts and unigenes in the S. salsa flower reference transcriptome. [file Table_2.docx]

**Table S2** Length distribution of transcripts and unigenes in the *S. salsa* flower reference transcriptome.

| Transcript length interval | 200-500bp | 500-1kbp | 1k-2kbp | >2kbp | Total |
| --- | --- | --- | --- | --- | --- |
| Number of transcripts | 136720 | 66261 | 59891 | 33013 | 295885 |
| Number of Genes | 64342 | 62456 | 59305 | 32970 | 219073 |
