# Supplementary material for: Salt-Enhanced Reproductive Development of Suaeda salsa L. Coincided With Ion Transporter Gene Upregulation in Flowers and Increased Pollen K+ Content
Source: Front Plant Sci. 2019 Mar 29;10:333. doi: 10.3389/fpls.2019.00333 (PMC6449877; doi:10.3389/fpls.2019.00333)
Supplement: TABLE S3 — Assembly statistics for the S. salsa flower reference transcriptome. [file Table_3.docx]

**Table S3** Assembly statistics for the *S. salsa* flower reference transcriptome.

|  | Min Length | Mean Length | Median Length | Max Length | N50 | N90 | Total Nucleotides |
| --- | --- | --- | --- | --- | --- | --- | --- |
| Transcripts | 201 | 919 | 557 | 16286 | 1509 | 369 | 271958919 |
| Genes | 201 | 1134 | 819 | 16286 | 1645 | 530 | 248500747 |

Note: N50 and N90 are the transcript lengths where 50% and 90% of the total nucleotides were assembled in transcripts longer than them, respectively.
