# Supplementary material for: Salt-Enhanced Reproductive Development of Suaeda salsa L. Coincided With Ion Transporter Gene Upregulation in Flowers and Increased Pollen K+ Content
Source: Front Plant Sci. 2019 Mar 29;10:333. doi: 10.3389/fpls.2019.00333 (PMC6449877; doi:10.3389/fpls.2019.00333)
Supplement: TABLE S4 — Gene function annotations in seven databases, including: NR, NT, KO, Swiss-prot, PFAM, GO, and KOG/COG, the gene number and the percentage of the total genes. [file Table_4.docx]

**Table S4** Gene function annotations in seven databases, including: NR, NT, KO, Swiss-prot, PFAM, GO, KOG/COG, the gene number and the percentage of the total genes.

|  | Number of Genes | Percentage (%) |
| --- | --- | --- |
| Annotated in NR | 132758 | 60.59 |
| Annotated in NT | 92443 | 42.19 |
| Annotated in KO | 49843 | 22.75 |
| Annotated in SwissProt | 94127 | 42.96 |
| Annotated in PFAM | 94085 | 42.94 |
| Annotated in GO | 94489 | 43.13 |
| Annotated in KOG | 32588 | 14.87 |
| Annotated in all Databases | 17891 | 8.16 |
| Annotated in at least one Database | 149297 | 68.14 |
| Total Unigenes | 219073 | 100 |
